# A systematic review and meta-analytic synthesis of the relationship between compulsory citizenship behaviors and its theoretical correlates

**DOI:** 10.3389/fpsyg.2023.1120209

**Published:** 2023-05-04

**Authors:** Bora Yildiz, Zeynep Kaptan, Tayfun Yildiz, Esengul Elibol, Harun Yildiz, Mustafa Ozbilgin

**Affiliations:** ^1^Department of Management, Faculty of Economics, Istanbul University, Istanbul, Türkiye; ^2^Department of Organizational Psychology, School of Business, Economics and Informatics, University of London-Birkbeck College, London, United Kingdom; ^3^Department of Management and Organization, Faculty of Economics and Administrative Sciences, Ardahan University, Ardahan, Türkiye; ^4^Department of Nursing, Faculty of Health Sciences, Istanbul Bilgi University, Istanbul, Türkiye; ^5^Department of International Trade, Omer Seyfettin Faculty of Applied Sciences, Bandirma Onyedi Eylul University, Balikesir, Türkiye; ^6^Brunel Business School, Organisations and People, College of Business, Arts and Social Sciences, Brunel University London, Uxbridge, United Kingdom

**Keywords:** compulsory citizenship behaviors, employees, meta-analysis, systematic review, CCBs

## Abstract

**Background:**

Compulsory citizenship behaviors (CCBs) are increasingly endorsed and expected of workers in contexts where managerial worker protections are low and performance demands on workers are high. Although studies on compulsory citizenship behaviors have shown a significant increase in recent years, the literature still lacks a comprehensive meta-analysis. To fill this gap the purpose of this study is to synthesize the collective outcomes of prior quantitative research on CCBs with the objective of identifying the factors linked to the concept and offering a primary reference for future researchers.

**Methods:**

Forty-three different correlates with CCBs were synthesized. The dataset of this meta-analysis consists of 53 independent samples with a sample size of 17.491, contributing to 180 effect sizes. PRISMA flow diagram and PICOS framework were used for the study design.

**Result:**

Results showed only gender and age were significant among demographic characteristics related to CCBs. Correlates between CCBs and counterproductive workplace behaviors, felt obligation, work-family conflict, organizational-based self-esteem, organizational cynicism, burnout, anger toward the organization, and work alienation were found as large. We also found turnover intention, moral disengagement, careerism, abusive supervision, citizenship pressure, job stress, facades of conformity, and feeling trusted to be moderately related to CCBs. Next, there was a small relationship between CCBs and social loafing. On the other hand, LMX, psychological safety, organizational identification, organizational justice, organizational commitment, job satisfaction, and job autonomy were found as significant deterrents of CCBs. These results suggest that CCBs flourish in contexts with low levels of worker protection and low road practices to people management.

**Conclusion:**

In sum, we found solid cumulative evidence that CCBs are a harmful and undesirable phenomenon for employees and organizations. Also, positive correlations of felt obligation, feeling trusted, and organization-based self-esteem with CCBs, showed that, contrary to general acceptance, positive factors could also cause CCBs. Lastly, we found CCBs as a dominant phenomenon in eastern culture.

## Introduction

Employee behaviors in the workplace play a critical role in the wellbeing of the organizations. To increse this contrubution, organizations or managers may want employees to contribute positively toward organizational outcomes (Luthans, 2002; Luthans and Avolio, 2009; Donaldson and Ko, 2010). In this context, employees are sometimes asked to do jobs beyond their formal job descriptions and to respond positively to such extra-curricular demands (Bowling, 2010; Bolino and Grant, 2016; Erdogan et al., 2020). One such voluntarily positive organizational behavior is organizational citizenship behaviors (OCB) which employees exhibit beyond their formal job descriptions (Organ, 1997; Podsakoff et al., 2000). When the relevant literature is examined, various positive organizational and managerial factors positively affect OCB (Organ and Ryan, 1995; Podsakoff et al., 2009; Chiaburu et al., 2011). However, the same factors also put pressure on the employees and force them to exhibit OCB forcibly, resulting behaviors are termed as CCBs (Vigoda-Gadot, 2006; Bolino et al., 2010; Peng and Zhao, 2012; Yildiz and Yildiz, 2015; Liu et al., 2017).

**Figure 1:**
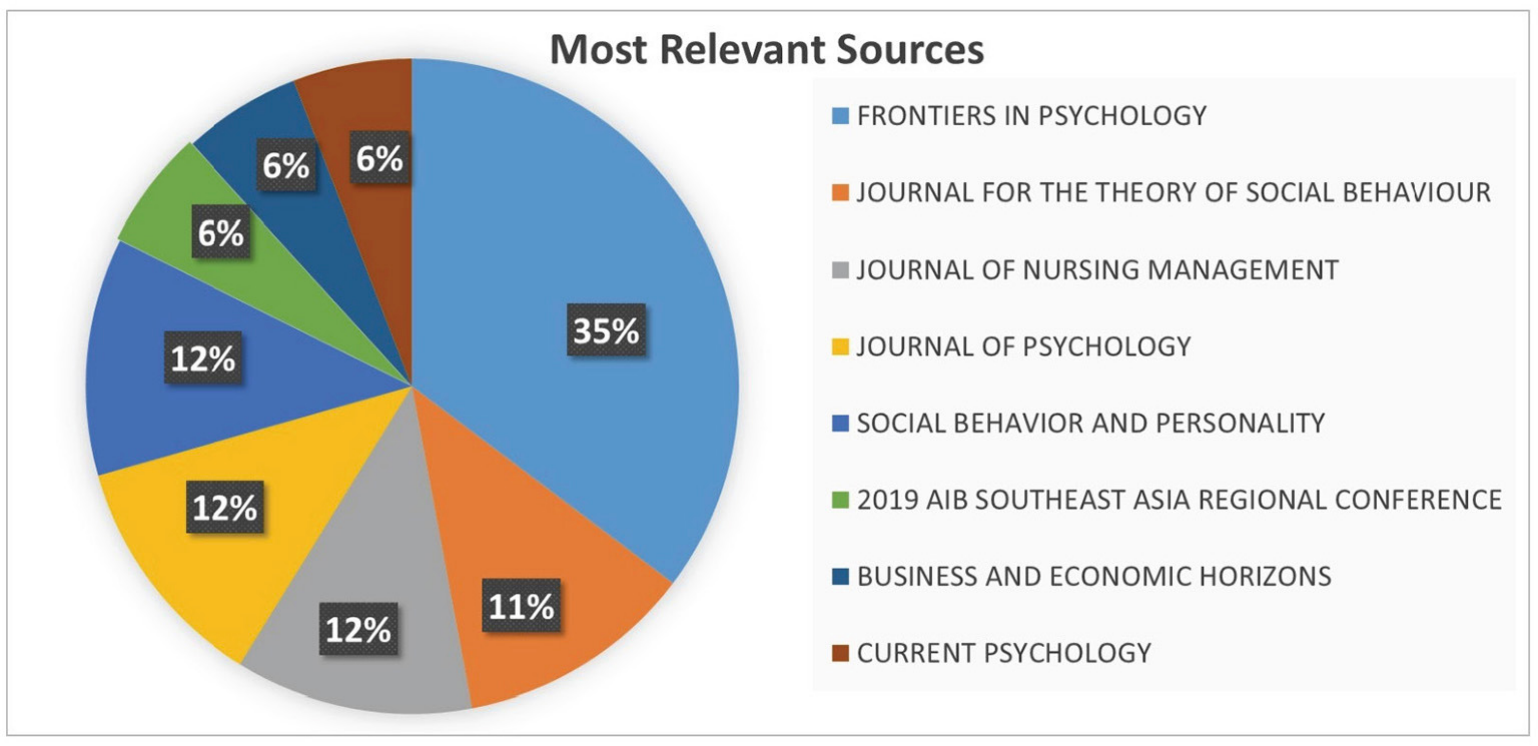
Most relevant sources of CCBs.

CCBs refer to involuntary extra-role behaviors that occur under pressure, not from the individual's goodwill (Vigoda-Gadot, 2007). According to the Automatic Data Processing (ADP) research institute reports, at the global level, employees work without pay for an average of 2 h per day (Tatum, 2022). Similarly, the same report pointed out that one out of every ten people works for at least 20 h a week for free. Plus, this rate has become remarkable, especially in the pre-pandemic period, with more than 5 million workers working more than 7.5 hours a day. This corresponds to 35 billion sterlings in the United Kingdom. Although CCBs are a global problem, it is more common in some countries with eastern cultures, such as Japan (Tatum, 2022), Turkey, and China. In these countries, respect creates a work environment where normative pressures can be exerted at work and in society (see Figure 1). In the organizational milieu, CCBs can appear in numerous forms; for example, employees' extra-curricular jobs at their discretion may become their duty over time. Another example of these behaviors is when the employee feels obliged to do jobs not in their job description for reasons such as helping others in need.

As Tatum (2022) pointed out, going above and beyond at work transformed “from exception to expectation” over time. The same author addressed many factors that play a pivotal role in this transformation. Some of these factors could be ordered as follows: career motivation, promotion expectations, decrease in unionization among employees, change in the structure and scope of work, blurriness of work boundaries, work engagement, and certain jobs or sectors (e.g., education, health, finance, consultancy, law). Also, cultural characteristics of countries, individual factors (e.g., ethnicity), situational factors (e.g., COVID-19), demographic characteristics (e.g., age, gender), intangible work outcomes, peer and managerial pressure, impression management, and the misconception that long working hours are directly proportional to productivity are among these factors.

**Figure 2:**
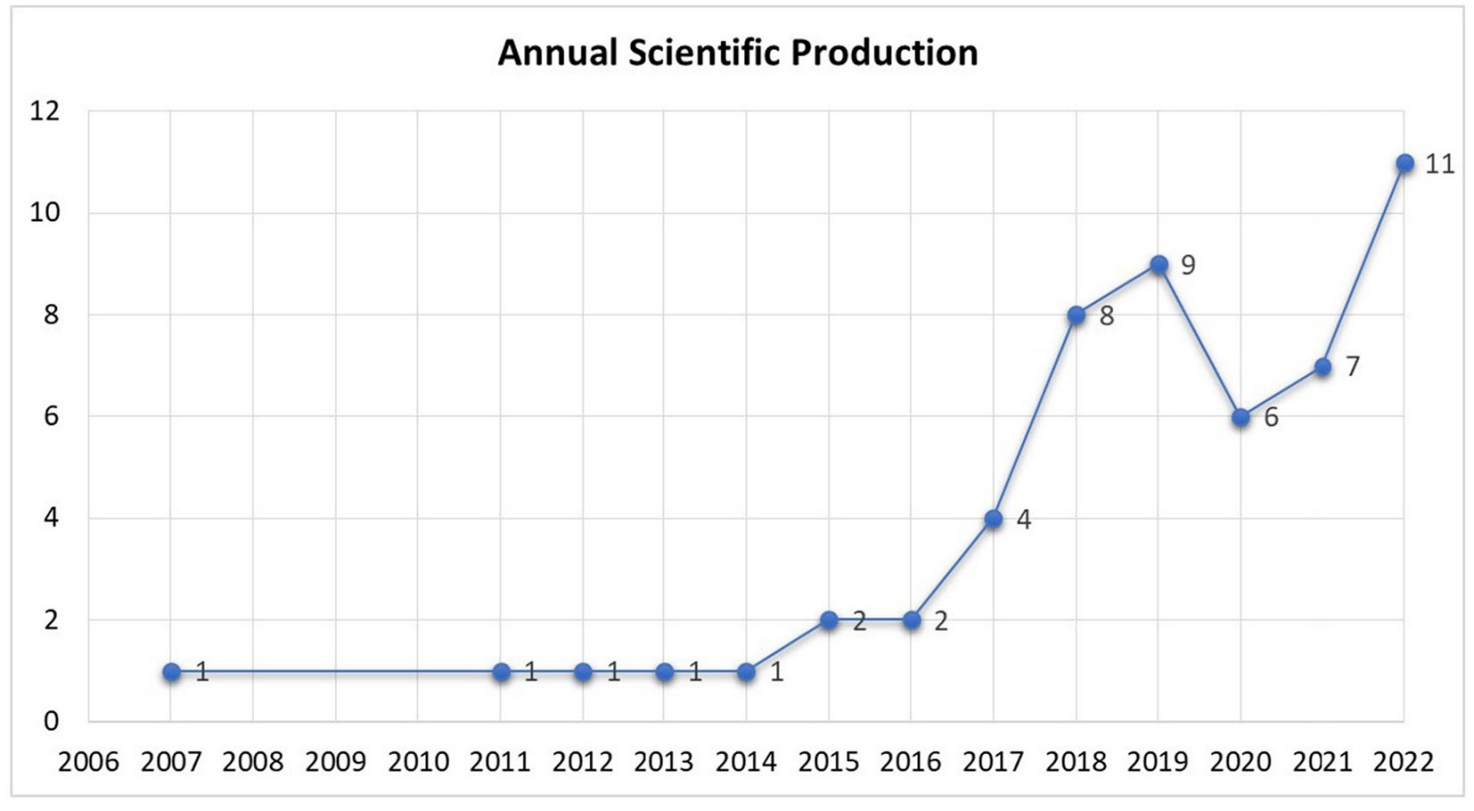
Annual scientific production of CCBs.

**Figure 3:**
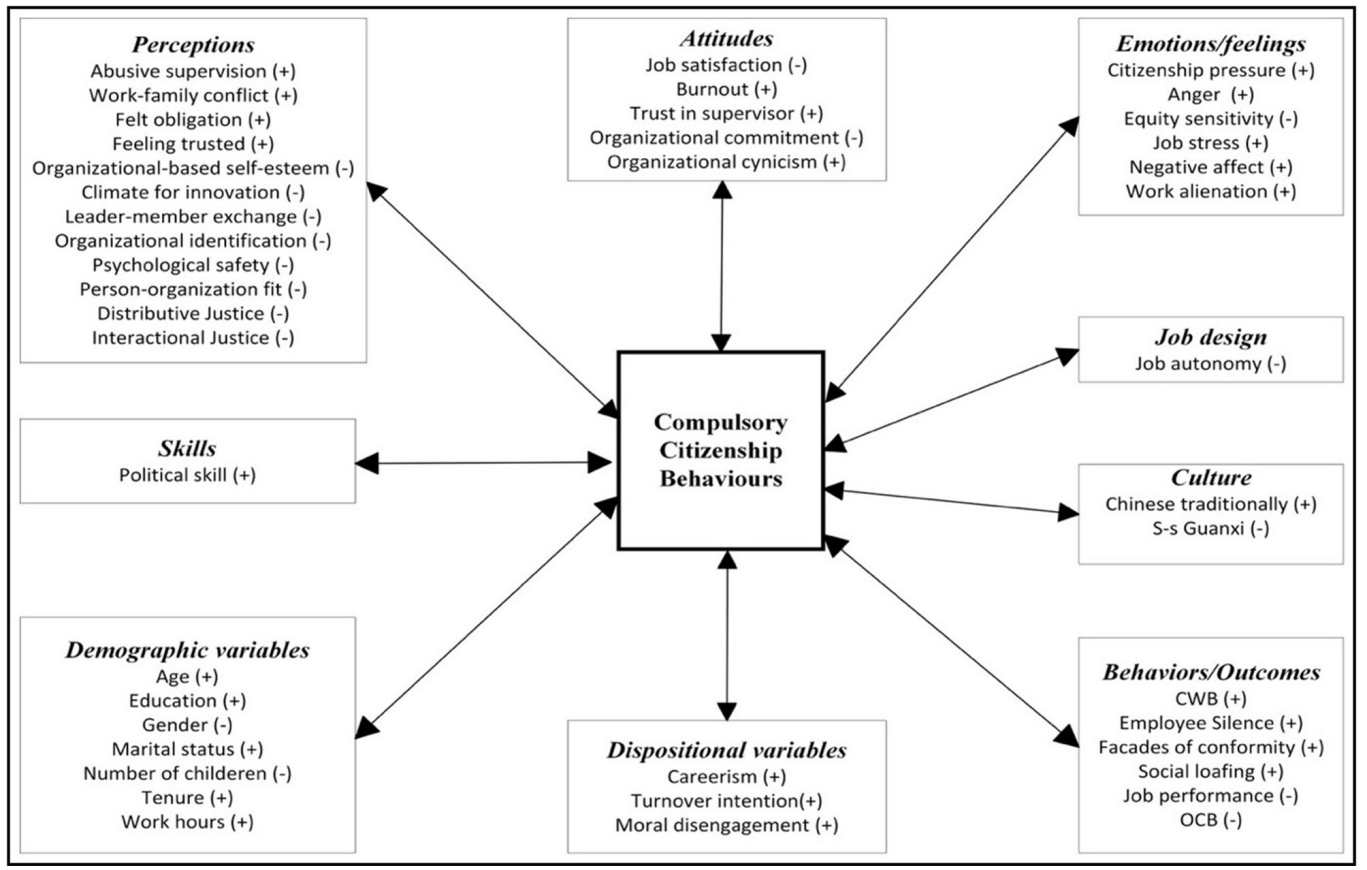
Overview of proposed nomological network of CCBs.

Since the last 16 years, especially in recent years, studies on the CCBs have been increasing at an accelerating rate (see Figure 2). Considering these devastating effects, researchers have identified many antecedents and consequences of CCBs based on different theoretical perspectives. The nomological network of the studies examined within the scope of this research is presented in Figure 3. Even if these behaviors supposedly benefit the organization in the short term, they reportedly cause significant harm to the employees and the organizations (e.g., CWB, Su et al., 2021; social loafing, Yildiz and Elibol, 2021; burnout, Pradhan and Gupta, 2021; job dissatisfaction, Che, 2015; lower level of job performance, Ahmadian et al., 2017; job stress, Chen et al., 2021; turnover intention, Yildiz and Elibol, 2021; work-family conflict, Chen et al., 2021).

On the other hand, there is some inconsistency in previous studies examining the relationships between CCB and its antecedents and consequences. Following variables are some examples of these inconsistencies: equity sensitivity (Guarino, 2016; Shu et al., 2018), Chinese traditionally (Peng and Zhao, 2012; Zhao et al., 2013), climate for innovation (Vigoda-Gadot, 2007; Ahmadian et al., 2017; Surucu, 2019), job stress (Surucu, 2019; Chen et al., 2021), negative affect (Bashir et al., 2019; He et al., 2020; Abukhait et al., 2023), political skill (Shu et al., 2018; Chen and Gao, 2020), OCB (Wang and Huang, 2019), and turnover intention (Yildiz and Ayaz Arda, 2018; Jin and Hahm, 2019).

Although some studies have conducted qualitative literature reviews that examine the concept in-depth and have addressed some of the above-mentioned inconsistencies (Vigoda-Gadot, 2006; Liu et al., 2019; Gencer Celik and Ongel, 2022; Gumussoy, 2022), no meta-analytic study has been found that empirically examines the cumulative results of these studies. One of the reasons behind this gap could be the complexities and challenges of quantifying the effect of factors such as measurement error and sampling error, which make the results inconsistent. Another reason could be the difficulty in calculating the cumulative effects due to the insufficient number of studies accumulated (Hunter and Schmidt, 2004). Despite the recent increase in CCBs studies, we think this gap in the literature limits the development of the field. Accordingly, this meta-analysis demonstrates which variables were associated with CCBs, and to what extent these variables were related to CCBs.

Considering the above, we not only provide a nomological network of CCBs but also shed light on many unresolved inconsistencies in the CCBs literature. Also, providing an analytical roadmap for potential researchers about what we already know about the CCBs and addressing potential gaps that need to be filled for the development of the field are other contributions of this article. By synthesizing 53 publications analytically, this paper highlights CCBs as the dark side of extra-role behaviors stemming from pressure at work and extends our understanding of CCBs. In this context, following the explanations and research purposes above, the research questions, based on the population, interventions, comparators, outcomes, and study designs (i.e., PICOS), that are tried to be answered in this study are as follows:

*RQ1:* What demographics are associated with CCBs?
*RQ2:* What is the overall effect size of the inconsistent results in the variables associated with CCBs, and in what direction?
*RQ3:* What variables are in the nomological network of CCBs?
*RQ4*: What are the neglected areas in research on CCBs?

## Literature review

### Compulsory citizenship behaviors

**Figure 4:**
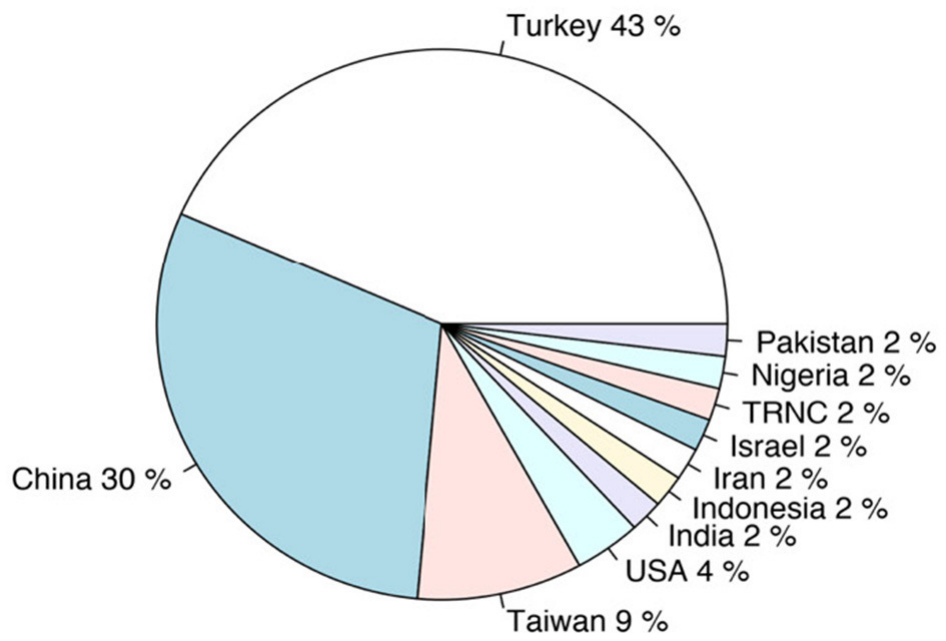
Distribution of the publications by countries.

Contemporary organizations face many environmental pressures, such as market competition, pressure from stakeholders, and keeping up with technological change (Frazier, 2013). These environmental pressures on organizations naturally cause an increase in expectations regarding the performance of employees. Thus, it is possible for employees in these organizations to be more involved in processes and to be with individuals who are more skilled and perform different tasks. Yet, in some countries, workers are protected by employment laws that prevent the imposition of extracurricular demands on workers. However, in some countries, employees may be exposed to normative pressures without supportive regulatory, union, or policy frameworks (Yildiz and Elibol, 2021; Yildiz et al., 2022a). As illustrated in Figure 4, the high frequency of publications on eastern cultures, such as Turkey and China, support this notion. Therefore, it is not unexpected that in recent years, managers in countries without supportive frameworks for workers could force employees to do CCBs, often without any monetary compensation. On the academic side, since the pioneering work of Vigoda-Gadot (2006, 2007) in introducing the concept of CCBs to the literature, studies on CCBs have shown a significant increase in recent years.

Further, as previously mentioned, many of the scientific publications on CCBs in the Web of Science index were published in Frontiers in Psychology (35%), followed by the Journal of Psychology (12%), Journal of Nursing Management (12%), and Social Behavior and Personality (12%). Considering the scientific journals above, both the publication of the articles in prestigious journals and the frequency of publication show that the concept of CCBs is topical and relevant.

Studies showed that CCBs are a common phenomenon seen in many industries (e.g., service, Ahmadian et al., 2017; finance, Alkan, 2015; education, Bashir et al., 2019; health, Yildiz and Elibol, 2021; manufacturing, Su et al., 2021; hospitality, Koksal, 2020). In the ongoing COVID-19 process, especially in the health sector, employees' sick leave, rest, and absenteeism have brought the employee workload to the fore. This situation caused employees to work beyond their potential and job descriptions (Shoja et al., 2020; Ebrahimi et al., 2021; Yildiz and Elibol, 2021; Reganata and Saputra, 2022). In line with these considerations, the results of a survey conducted by the National Health Service in England in January 2022, indicated that 75% of the healthcare professionals exposed to exhibit CCBs without pay and out of their goodwill (Tatum, 2022). Although the birth of the concept (CCBs) is not related to COVID-19 (Tatum, 2022), the pandemic, a situational factor, fostered more CCBs. Excessive work demands, unsatisfactory paid sick leave benefits, and employees' fear of losing jobs are the causes of this result (Paglinawan, 2021; Sinclair et al., 2021; Hassanie et al., 2022; Luan et al., 2022). In parallel with this idea, COVID-19 has not only reinforced the existing negative consequences of CCBs but also has transformed the generally accepted positive associations into negative ones. For example, a recent meta-analytic study, which examined the counterintuitive effects of COVID-19 on employees, showed that the positive and significant link between work engagement and job satisfaction has turned into a negative and non-significant form during the ongoing pandemic (Yildiz et al., 2022b).

**Figure 5:**
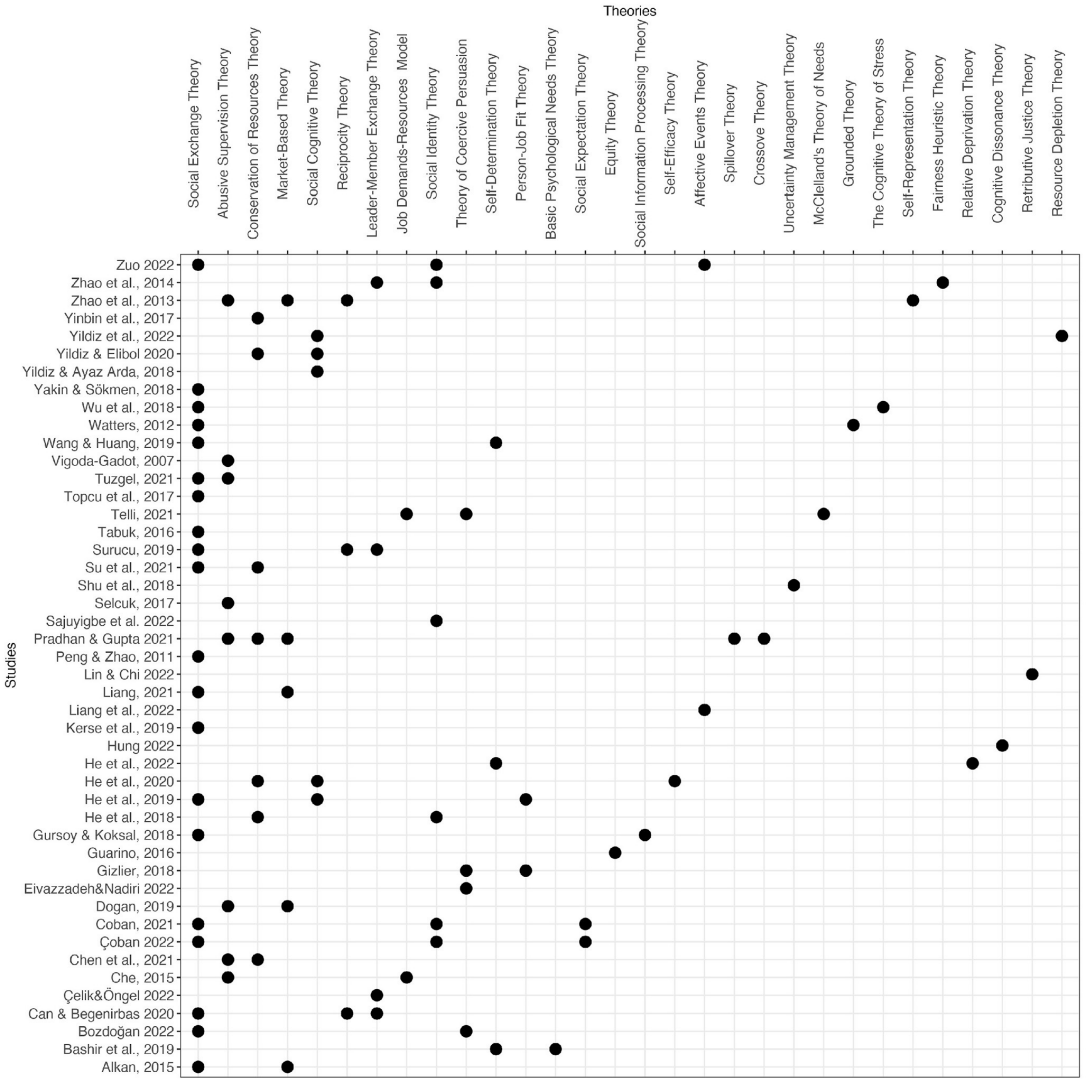
Specific theories used by synthesized publications.

CCBs, which were formerly often associated with dark leadership styles such as abusive supervision and authoritarian leadership (Shu et al., 2018; Wang and Huang, 2019; Pradhan and Gupta, 2021), throughout the COVID-19 pandemic, have transformed a phenomenon seen in almost all countries (Yildiz et al., 2022a). However, the fact that CCBs are common in eastern cultures remain the same. To prevent and manage CCBs, researchers benefited from different theoretical perspectives that shed light on many studies (see Figure 5). Accordingly, previous studies developed their research model primarily by grounding on the social exchange theory (SET), abusive supervision theory (AST), and conservation of resources theories (COR) (e.g., Zhao et al., 2013; Wu et al., 2018; Wang and Huang, 2019; Chen et al., 2021).

When the nomologic network of the CCBs was examined, four research trends drew attention. Focusing CCBs' destructive effects on employees' psychological sources, emotions, cognitive processes, and behaviors are one of them. Understanding the employees' reactions to CCBs is the second research trend in CCBs studies. The third one is looking at CCBs as an outcome of positive drivers, such as feeling trusted by the manager. Finally, as mentioned above, the last research trend is that CCBs are an extension of dark managerial or leadership styles.

On the other hand, when the correlations are categorized based on classical theories, past researchers have examined the relations of many perceptions (e.g., supervision, Pradhan and Gupta, 2021), attitudes (e.g., trust in supervisor), behaviors (e.g., counterproductive workplace behaviors, Su et al., 2021), feelings/emotions (e.g., job stress), dispositional variables (e.g., turnover intention) and cultural factors (e.g., S-s Guanxi, Wu et al., 2018) with CCBs. The conceptual definitions of studied variables are presented in Table 1. Despite the accumulation of the related correlates of CCBs, there are some inconsistencies in the current literature. While some studies found a positive relationship with CCBs, others found a negative. Some of these variables are as follows; equity sensitivity (Guarino, 2016) climate for innovation (Surucu, 2019), and OCB (Wang and Huang, 2019). One potential reason for these inconsistencies could be explained by statistical artifacts (e.g., sampling or measurement errors) (Schmidt and Hunter, 2014). Further, as Luan et al. (2022) addressed, another reason could be the potential opposite mechanisms between the above-mentioned variables. To minimize these inconsistencies, new empirical research needs to be accumulated (particularly moderator variables) and the accumulated research pile should be updated with new meta-analyses.

**Table 1:** Conceptual definitions and classification of study variables.

**Variable**	**Definition**
**Ability/skills**	
Political skill (PL)	PL refers to “the ability to effectively understand others at work, and to use such knowledge to influence others to act in ways that enhance one's personal and/or organizational objectives” (Ahearn et al., 2004, p. 311).
**Job characteristics**	
Job autonomy (JA)	JA means “the degree to which employees can control and decide on their own methods of work, work arrangements, and work standards” (Breaugh, 1985, p. 556).
**Dispositional variables**	
Careerism	Careerism refers to “the propensity to pursue career advancement through non-performance-based means” (Feldman and Weitz, 1991, p. 238).
Turnover intention (TI)	TI means “a conscious and deliberate willfulness to leave the organization” (Tett and Meyer, 1993, p. 262).
Moral disengagement (MD)	MD refers to “a conglomeration of self-regulatory processes that allow people to act unethical without feeling bad” (Knoll et al., 2016, p. 65).
**Emotions/moods/feelings**	
Citizenship pressure (CP)	CP refers to “a specific job demand in which an employee feels pressured to perform OCBs” (Bolino et al., 2010, p. 836).
Anger toward organization (ATO)	*ATO* means “extreme displeasure, hostility, indignation, or exasperation toward someone or something” (Berube, 1985, p. 109).
Equity sensitivity (ES)	ES states that “individuals subscribe to the norm of equity and feel distress when either overrewarded or under rewarded” (Huseman et al., 1987, p. 225).
Job stress (JS)	JS refers to “a particular individual's awareness or feeling of personal dysfunction as a result of perceived conditions or happenings in the work setting” (Parker and Decotiis, 1983, p. 161).
Negative affect (NA)	NA means “a general dimension of subjective distress and unpleasurable engagement that subsumes a variety of aversive mood states, including anger, contempt, disgust, guilt, fear, and nervousness” (Watson et al., 1988, p. 1063).
Work alienation (WI)	WI refers to “a state (or feeling) in which the job is external to the individual” (Sarros et al., 2002, p. 287).
**Perceptions**	
Abusive supervision (AS)	AS refers to “subordinates' perceptions of the extent to which supervisors engage in the sustained display of hostile verbal and nonverbal behaviors, excluding physical contact” (Tepper, 2000, p. 178).
Climate for innovation (CFI)	CFI states “a multi-dimensional context which includes the intention to be innovative, the infrastructure to support innovation, operational level behaviors necessary to influence a market and value orientation, and the environment to implement innovation” (Dobni, 2008, p. 540).
Distributive justice (DJ)	DJ refers to “the fairness of rewards (or punishments)” (Alexander and Ruderman, 1987, p. 177).
Felt obligation (FO)	FO means “a prescriptive belief regarding whether one should care about the organization's well-being and should help the organization reach its goals” (Eisenberger et al., 2001, p. 42).
Feeling trusted (FT)	FT states that “a leader's humble behaviors become useful behavioral cues for the employee that he or she is perceived as trust-worthy (e.g., competent, and benevolent) by the leader” (Cho et al., 2021, p. 172).
Interactional justice (IJ)	IJ refers to “the interpersonal treatment they receive at the hands of organizational decision makers” (Cropanzano et al., 2002, p. 325).
Leader-member exchange (LMX)	LMX describes “the tendency of managers to develop different types of relationships with their employees, leading to the use of different management styles depending on the employee” (Liden et al., 1997).
Organizational identification (OI)	OI states “a perceived oneness with an organization and the experience of the organization's successes and failures as one's own” (Mael and Ashforth, 1992, p. 103).
Organizational-based self-esteem (OBSE)	OBSE refers to “the degree to which employees perceive themselves as important, meaningful, effectual and worthwhile within their employing organization” (Pierce et al., 1989, p. 643).
Psychological safety (PS)	PS describes “people's perceptions of the consequences of taking interpersonal risks in a particular context such as a workplace” (Edmondson and Lei, 2014, p. 23).
P-O fit	P-O fit states “the compatibility between people and organizations that occurs when: (a) at least one entity provides what the other needs, or (b) they share similar fundamental characteristics, or (c) both” (Kristof, 1996, p. 4).
Work-family conflict (WFC)	WFC means “one type of interrole conflict in which role pressures from the work and family domains involve some level of mutual incompatibility” (Nielson et al., 2001, p. 366).
**Attitudes**	
Job satisfaction (JS)	JS means “the pleasurable emotional state resulting from the appraisal of one's job as achieving or facilitating the achievement of one's job values” (Locke, 1969, p. 316).
Burnout	Burnout refers to “an internal and psychological experience involving feelings, attitudes, motives, and expectations; and that it is a negative experience for the individual, in that it concerns problems, distress, discomfort, dysfunction, and/or negative consequences” (Maslach et al., 2009, p. 89).
Trust in supervisor (TS)	TS means “the willingness of a party to be vulnerable to the actions of another party based on the expectation that the other will perform a particular action important to the trustor, irrespective of the ability to monitor or control that other party” (Mayer et al., 1995, p. 712).
Organizational commitment (OCo)	OCo states “a psychological state that (a) characterizes the employee's relationship with the organization, and (b) has implications for the decision to continue or discontinue membership in the organization” (Meyer and Allen, 1991, p. 67).
Organizational cynicism (OCy)	OCy refers to “a negative attitude toward one's employing organization, comprising three dimensions: (1) a belief that the organization lacks integrity; (2) negative affect toward the organization; and (3) tendencies to disparaging and critical behaviors toward the organization that are consistent with these beliefs and affect” (Dean et al., 1998, p. 345).
**Behaviors/outcomes**	
Counterproductive workplace behavior (CWB)	CWB states “any intentional behavior on the part of an organizational member viewed by the organization as contrary to its legitimate interests” (Sackett, 2002, p. 5).
Employee silence (ES)	ES defines “a person's withholding of genuine expression about behavioral, cognitive and/or affective evaluations of organizational circumstances to persons perceived capable of effecting change or redress” (Harlos, 2016, p. 346).
Creating facades of conformity (CFC)	CFC refers to “a move from one's position to a contradictory behavior or belief as a result of real or imagined group pressure” (Hewlin, 2009, p. 728).
Job performance (JP)	JP states “the total expected value to the organization of the discrete behavioral episodes that an individual carries out over a specified time period” (Motowidlo and Kell, 2012, p. 39).
Organizational citizenship behavior (OCB)	OCB refers to “individual behavior that is discretionary, not directly or explicitly recognized by the formal reward system, and that in aggregate promotes the effective functioning of the organization” (Organ, 1988, p. 4).
Social loafing (SL)	SL defines “the reduction in motivation and effort when individuals work collectively compared with when they work individually and coactively” (Karau and Williams, 1993, p. 681).
**Chinese culture**	
Chinese traditionality (CT)	CT states “an individual's endorsement of hierarchical role relationships as defined by the five cardinal relationships (called wu-lun) in Confucianism (i.e., emperor-subject, father-son, husband-wife, older brother-younger brother, and friend-friend), reflecting the cultural dimension of employees' submission to authority” (Wang et al., 2010, p. 150).
Supervisor-subordinate guanxi (SSG)	SSG refers to “a personal relationship between a supervisor and a subordinate developed largely from nonwork related social interactions that might extend into the workplace” (Guan and Frenkel, 2019, p. 1753).

## The present study

In this meta-analytic review, we have three primary objectives. First, we tried to determine the current nomological network of the CCBs. Because the studies on CCBs were conducted in different disciplines and associated with different concepts, this developing process of the CCBs has expanded over the years. Therefore, many significant findings about the nomological network of CCBs may have been overlooked by past researchers. Second, we tried to reinforce the current research findings in the literature. Despite the growing literature on CCBs, the results of some studies are mixed. While some studies determined significant, positive, or negative correlations between CCBs and studied variables, others found the exact opposite. Lastly, this study provides an analytic framework that summarizes and cumulates the existing knowledge for further research. Hence, this study can help give a useful direction to the current literature and fill the promising gaps that have been neglected until now.

The flow of the research is presented under the following headings to achieve the three main objectives mentioned above. The method section contains the following subheadings;, literature review and inclusion criteria (formulation of the research question with PICOS framework and PRISMA flow diagram), information about scales used to measure CCBs, meta-analytical procedure, and characteristics of included studies (e.g., country, method, sector, data collection time, gender dominance, theoretical basis). Finally, results were presented under two main sub-titles: findings related to demographic variables and findings related to research variables.

## Methods

### Literature search and inclusion criteria

Recent systematic reviews and meta-analyses operated the PICOS framework to standardize the research design process (Koh et al., 2020; Yildiz et al., 2022b). In this respect, the PICOS framework was used for research question formulation. Accordingly, the PICOS framework is as follows: population (P): employees, interventions (I): not applicable, comparators (C): individual effect sizes vs. overall effect sizes, outcomes (O): effect sizes of the examined relations, and study design (S): a correlational meta-analysis.

**Figure 6:**
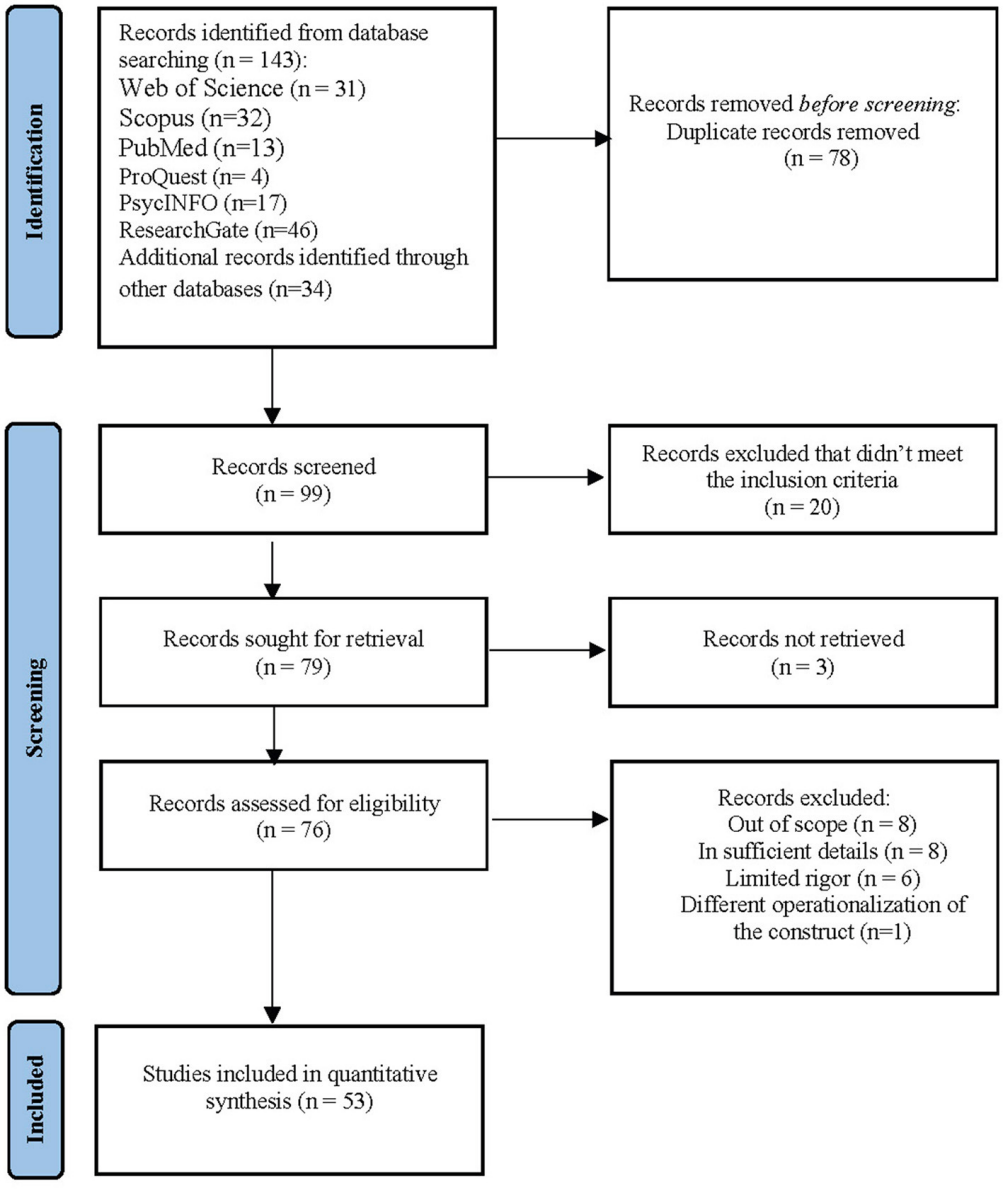
PRISMA flow diagram.

To determine the studies that investigated the correlation between CCBs and other variables following databases were searched (see Figure 6): Web of Science (WoS), Scopus, PsycINFO, ProQuest, PubMed, ResearchGate, Google Scholar, and Yok Tez (Turkish Council of Higher Education Thesis Center). EndNote (v. 20) software was used as a reference management tool. The following keywords were used to search databases: (“*Compulsory Citizenship Behav” OR “Compulsory Organizational Citizenship” OR “Citizenship Pressure” OR “Compulsory Citizenship”) AND (“Employee” OR “Work” OR “Job” OR “Organization”*). The main reason we use these databases is that the mentioned databases pass a rigorous peer-review process for the publications they have published. On the other hand, Google Scholar, ProQuest, and Yok Tez databases were searched to determine the gray literature that has not yet been published (e.g., dissertations, conference papers, and works in progress). All databases were searched with the same keywords. The search process of the databases was terminated on 31 October 2022. The inclusion criteria of this meta-analysis are as follows:

- Studies should be published in English or Turkish language.
- The included studies should report a correlation coefficient between CCBs and any other variable or have sufficient statistics that enable researchers to calculate the correlation coefficient and, in turn, effect sizes (e.g., sample size and *t*-test value).
- The samples of the studies should consist of employees in non-managerial positions.
- Studies should refer to Vigoda-Gadot's CCBs concept definition.
- Studies should use valid and reliable scales.
- Studies should be self-reported by employees, not by supervisors or managers.

According to the inclusion criteria, in total, 53 studies were coded by two authors. Next, to test inter-rater reliability, one study was randomly selected from the publication pool and recorded by these two coders. Accordingly, inter-rater reliability among the three authors was high (Cohen's Kappa coefficient = 0.90). Consequently, 53 correlates with compulsory citizenship behaviors, and 180 effect sizes were utilized to analyze CCBs.

### Measures of compulsory citizenship behaviors

All the included studies (*k* = 53) measured CCBs with Vigoda-Gadot's (2007) five-item scale. The scale asked respondents to report how often they are exposed to displaying CCBs. Recent studies have tested and provided reliability and validity evidence of the Vigoda-Gadot's scale by using clasical test and item response theories (Yildiz, 2022). In Vigoda-Gadot's (2007) study, Cronbach's alpha internal consistency of the scale (α) was determined as 0.83. On the other hand, the range of α coefficient in the included studies varies from 0.65 to 0.96 (mean = 0.86 ± 0.07, median = 0.87).

### Meta-analytic procedure

In this study, to analyze data, R Statistical language was used (version 4.2.0) (R Core Team, 2023). As well as individual effect sizes, other study characteristics such as authors, years, countries, sample sizes, sectors, type (published/unpublished), presence of outlier management, scale, and reliability coefficients of the scale were coded into the coding file to be used as potential moderators (see Supplementary Data).

This meta-analysis was run based on Schmidt and Hunter's (2014) meta-analytic approach. Firstly, the overall effect sizes of the relations among the variables were calculated. Schmidt and Hunter (2014) emphasize that various biases, measurement errors, and sampling errors in cumulating effect sizes in a meta-analysis may have effects. Unlike their book published in 1990 (Hunter and Schmidt, 1990), Schmidt and Hunter (2014) suggest various statistical artifacts to correct the influences of sampling, measurement errors, and biases caused by researchers on meta-analysis. Accordingly, artifact distributions of the average reliability estimates were presented in Table 2. To test whether there was a publication bias in all meta-analyses, Egger's regression test (Egger et al., 1997) was used, and no publication bias was found (*p* < 0.05). Further, to test the file drawer problem, a fail-safe N analysis was conducted for each overall effect size (Rosenthal, 1979). Finally, although moderator analysis is required to explain the heterogeneity in effect sizes (Yildiz et al., 2021), because of the limited number of studies, moderator analysis could not be conducted to elucidate the heterogeneity in effect sizes (Chamberlin et al., 2017; Mercado et al., 2017; Hao et al., 2022; Ogunfowora et al., 2022).

**Table 2:** Reliability artifact distributions.

**Variables**	***k***	****r*_*xx*_***	**SD*r_*xx*_***	***M* $\sqrt{r}$ _*xx*_**	** $SD\sqrt{r}$ _*xx*_**
Abusive supervision	4	0.93	0.02	0.96	0.01
Anger toward organization	2	0.81	0.11	0.90	0.06
Burnout	10	0.86	0.07	0.93	0.04
Careerism	3	0.86	0.07	0.92	0.04
Chinese traditionality	2	0.89	0.05	0.94	0.03
Citizenship pressure	2	0.85	0.02	0.92	0.01
Climate for innovation	3	0.89	0.04	0.94	0.02
Compulsory citizenship behaviors	53	0.86	0.07	0.93	0.04
Counterproductive workplace behaviors	3	0.91	0.04	0.95	0.02
Employee silence	2	0.83	0.03	0.91	0.02
Equity sensitivity	3	0.91	0.04	0.95	0.02
Facades of conformity	2	0.83	0.00	0.91	0.00
Feeling trusted	2	0.90	0.07	0.95	0.03
Felt obligation	2	0.90	0.06	0.95	0.03
Job autonomy	2	0.96	0.02	0.92	0.01
Job performance	4	0.90	0.05	0.95	0.03
Job satisfaction	6	0.85	0.08	0.92	0.04
Job stress	8	0.89	0.06	0.94	0.03
Leader-member exchange	6	0.87	0.06	0.93	0.03
Moral disengagement	3	0.85	0.06	0.92	0.03
Negative affect	3	0.82	0.04	0.91	0.02
Organizational based self esteem	2	0.90	0.06	0.95	0.03
Organizational citizenship behaviors	5	0.87	0.04	0.93	0.02
Organizational commitment	3	0.79	0.06	0.89	0.04
Organizational cynicism	4	0.89	0.04	0.95	0.02
Organizational identification	3	0.86	0.04	0.93	0.02
Organizational justice					
Distributive justice	2	0.90	0.06	0.95	0.03
Interactional justice	2	0.86	0.01	0.93	0.01
Person-organization fit	2	0.90	0.03	0.95	0.02
Political skill	2	0.77	0.12	0.87	0.07
Psychological safety	3	0.91	0.05	0.95	0.02
S-s guanxi	2	0.83	0.06	0.91	0.03
Social loafing	3	0.90	0.01	0.95	0.01
Trust in supervisor	2	0.90	0.07	0.95	0.03
Turnover intention	12	0.87	0.05	0.93	0.03
Work alienation	2	0.89	0.01	0.94	0.01
Work-family conflict	4	0.88	0.05	0.94	0.03

*k*, number of independent reliability estimates in distribution; $\bar{r}$_*xx*_, average reliability estimate; *SDr*_*xx*_, standard deviation of reliability estimates; *M*$\sqrt{r}$_*xx*_, average of the square roots of reliability estimates; $SD\sqrt{r}$_*xx*_, standard deviation of the square roots of the reliability estimate.

### Study characteristics

In this meta-analytic review, we synthesized 53 (41 of which were published, and the remaining 12 were gray literature) quantitative publications related to CCBs. The sum of the sample sizes of the synthesized studies (*k* = 53) is 17.197 (324 ± 154). As illustrated in Figure 4, most synthesized studies were conducted in Turkey (43%) and China (32%). Most of the included studies have a cross-sectional research design (72%).

While eight studies did not report where the data were collected, half of the remaining 24 studies' data were collected while at work. The other 21 studies' data were collected outside of work or by online data collection method. When the included studies' gender distribution was evaluated, four studies did not make a specific explanation about the distribution of the sample according to gender. In comparison, 25 of the remaining 49 studies sample consists of predominantly male participants, and the other 24 studies consist of mostly female participants. Also, when the sectoral distribution of the included studies is evaluated, it is noteworthy that while the sectoral information of the sampling was not given in 15 studies, most of the remaining studies (*k* = 37) were conducted in the service (28.6%), manufacturing (25%), health (17.9%), education (14.3%), and finance (14.3%) sectors, respectively (see Supplementary Data).

As illustrated in Figure 5, most of the included studies (*k* = 46) are based on at least one theory. Specifically, CCBs-related studies are primarily based on the SET (*k* = 20), AST (*k* = 8), COR (*k* = 7), social identity theory (*k* = 6), and market-based theory (*k* = 5), respectively. The points in Figure 5 are located at the intersection of the study name on the horizontal axis and the theories used in that study on the vertical axis. Accordingly, the points on the row where each study is located show that the theory/theories corresponding to that point on the vertical axis were employed to elucidate the links between CCBs and other study variables.

## Results

Table 3 represents the meta-analytic findings among CCBs and demographic and other research variables. Each synthesized study cluster by variables is provided in Supplementary Data. There is a small and statistically significant relationship between CCBs and gender [*r* = −0.03, ρ = −0.04, 90% CI (−0.08, −0.01)], suggesting that male workers exposed to display more CCBs. The number of children also displayed a small and statistically significant correlation between CCBs [*r* = −0,04, ρ = −0.05, 90% CI (−0.09, −0.01)], signifying that those employees with fewer children exhibit more CCBs. However, age, education, tenure, marital status, and work hours demonstrated negligible and non-significant relationships with CCBs (90% CI include zero, see Table 3).

**Table 3:** Meta-analytic findings for demographic and research variables.

**Variables**	***k***	***N***	** $\bar{r}$ **	**SD_r_**	**SD_res_**	**SE of $\bar{r}$**	**ρ**	**SD*_ρ_***	**90% CI*_ρ_***	**80% CV*_ρ_***
Age^a^	19	6,456	0.02	0.06	0.02	0.01	0.02	0.03	−0.01, 0.05	−0.02, 0.06
Gender	18	6,339	−0.03	0.06	0.03	0.01	−0.04	0.03	–**0.08**, –**0.01**	−0.09, 0.00
Education	14	5,196	0.02	0.10	0.09	0.03	0.02	0.11	−0.04, 0.09	−0.12, 0.17
Tenure	10	3,426	−0.02	0.10	0.09	0.03	−0.02	0.11	−0.11, 0.06	−0.17 0.12
Marital status	5	2,100	0.03	0.06	0.04	0.03	0.04	0.05	−0.04, 0.12	−0.03, 0.11
Work hours	5	1,870	0.07	0.11	0.10	0.05	0.09	0.12	−0.05, 0.22	−0.07, 0.24
Number of children	3	1,323	−0.04	0.02	0.00	0.01	−0.05	0.00	–**0.09**, –**0.01**	−0.05,−0.05

*k*, number of independent samples included in the analysis; *N*, total sample size; $\bar{r}$, mean sample-sizeweighted observed correlation; SD*r*, standard deviation of mean sample-size-weighted correlations; SD_res_, residual standard deviation; SE $\bar{r}$, standard error of $\bar{r}$,; ρ, true-score correlation; 90% CIρ, 90% confidence interval of ρ; 80% CVρ, 80% credibility values of ρ.

^a^0 = male, 1 = female. Bold values indicate statistically significant meta correlation coefficients.

As presented in Table 4, meta-analyses of the related variables were classified into eight sub-categories: job design, skills, dispositional variables, perceptions, attitudes, behaviors/outcomes, feelings/emotions, and culture. In Table 4, in which the meta-analysis results regarding study variables are presented, the overall effect sizes are listed from large to small according to the degree of effect size. The relatively high effect sizes suggest that the variable is more strongly associated with CCBs compared to other variables.Therefore, the factors with the highest correlation in each sub-category are presented in the first place.

**Table 4:** Meta-analytic results.

**Variables**	***k***	***N***	** $\bar{r}$ **	**SD*_*r*_***	**SD_res_**	**SE of $\bar{r}$**	**ρ**	**SD*_ρ_***	**90% CI*_ρ_***	**80% CV*_ρ_***
**Dispositional variables**										
Turnover intention	12	4,354	0.34	0.17	0.17	0.05	0.43	0.21	**0.32, 0.55**	0.16, 0.70
Moral disengagement	3	1,112	0.33	0.12	0.11	0.07	0.42	0.13	**0.16, 0.67**	0.25, 0.59
Careerism	3	942	0.29	0.08	0.06	0.05	0.37	0.08	**0.19, 0.55**	0.27, 0.48
**Skills**										
Political skill	2	535	−0.08	0.10	0.08	0.07	−0.10	0.10	−0.67, 0.46	−0.23, 0.02
**Job design**										
Job autonomy	2	518	−0.05	0.01	0.00	0.01	−0.06	0.00	**−0.12**, **−0.01**	−0.06, −0.06
**Perceptions**										
Felt obligation	2	858	0.59	0.00	0.00	0.00	0.75	0.00	**0.74, 0.77**	0.75, 0.75
Work-family conflict	4	1,232	0.57	0.12	0.11	0.06	0.72	0.13	**0.54, 0.90**	0.55, 0.90
Organization-based self-esteem	2	858	0.49	0.05	0.02	0.04	0.62	0.03	**0.31, 0.93**	0.59, 0.65
Feeling trusted	2	858	0.38	0.01	0.00	0.01	0.49	0.00	**0.41, 0.58**	0.49, 0.49
Abusive supervision	4	1,409	0.32	0.17	0.16	0.08	0.37	0.18	**0.15, 0.60**	0.14, 0.61
Climate for innovation	3	1,143	−0.04	0.19	0.19	0.11	−0.05	0.24	−0.47, 0.37	−0.35, 0.26
Interactional justice	2	1,313	−0.13	0.01	0.00	0.01	−0.15	0.00	–**0.20**, –**0.10**	−0.15, −0.15
Distributive justice	2	1,072	−0.23	0.05	0.02	0.03	−0.30	0.03	–**0.58**, –**0.02**	−0.33, −0.27
Organizational identification	3	1,015	−0.24	0.12	0.10	0.07	−0.31	0.13	–**0.56**, –**0.06**	−0.48, −0.05
Psychological safety	3	1,429	−0.28	0.04	0.00	0.02	−0.32	0.00	–**0.40**, –**0.24**	−0.32, −0.32
Leader-member exchange	6	1,850	−0.37	0.09	0.07	0.04	−0.48	0.09	–**0.57**, –**0.38**	−0.60, −0.36
Person-organization fit	2	598	−0.49	0.14	0.13	0.10	−0.63	0.16	−1.44, 0.17	0.42, 0.84
**Attitudes**										
Organizational cynicism	4	964	0.60	0.08	0.06	0.04	0.77	0.07	**0.65, 0.90**	0.69, 0.73
Burnout	10	3,430	0.55	0.11	0.10	0.03	0.71	0.12	**0.63, 0.79**	0.55, 0.87
Trust in supervisor	2	858	0.07	0.04	0.00	0.03	0.09	0.00	−0.16, 0.35	0.09, 0.09
Organizational commitment	3	895	−0.20	0.09	0.07	0.05	−0.26	0.09	–**0.46**, –**0.06**	−0.37, −0.14
Job satisfaction	6	950	−0.33	0.09	0.05	0.04	−0.43	0.06	–**0.52**, –**0.33**	−0.50, −0.35
**Behaviors**										
Employee silence	2	767	0.49	0.20	0.19	0.14	0.63	0.24	−0.51, 1.78	0.32, 0.95
CWB	3	984	0.47	0.13	0.12	0.07	0.60	0.15	**0.33, 0.88**	0.41, 0.79
Facades of conformity	2	1,011	0.37	0.00	0.00	0.00	0.48	0.00	**0.45, 0.51**	0.48, 0.48
Social loafing	3	758	0.24	0.14	0.13	0.08	0.28	0.16	**0.01, 0.55**	0.09, 0.44
OCB	5	1,728	0.02	0.27	0.26	0.12	0.02	0.30	−0.27, 0.32	−0.36, 0.41
Job performance	4	1,478	−0.09	0.09	0.08	0.05	−0.11	0.10	−0.25, 0.03	−0.24, 0.01
**Feelings/emotions**										
Work alienation	2	686	0.50	0.04	0.00	0.03	0.64	0.00	**0.39, 0.90**	0.64, 0.64
Anger toward organization	2	365	0.41	0.02	0.00	0.01	0.52	0.00	**0.43, 0.61**	0.52, 0.52
Citizenship pressure	2	668	0.36	0.03	0.00	0.02	0.46	0.00	**0.29, 0.64**	0.46, 0.46
Job stress	8	2,609	0.32	0.35	0.35	0.12	0.42	0.44	**0.11, 0.72**	−0.15, 0.98
Negative affect	3	861	0.15	0.12	0.11	0.07	0.19	0.13	−0.07, 0.46	0.02, 0.36
Equity sensitivity	3	660	0.01	0.04	0.00	0.02	0.02	0.01	−0.06, 0.09	0.01, 0.01
**Chinese culture**										
Chinese traditionality	2	884	0.05	0.23	0.23	0.16	0.07	0.29	−1.25, 1.39	−0.30, 0.43
S-s guanxi	2	617	−0.09	0.04	0.00	0.03	−0.12	0.00	−0.35, 0.11	−0.12, −0.12

*k*, number of independent samples included in the analysis; *N*, total sample size; $\bar{r}$, mean sample-sizeweighted observed correlation; SD*r*, standard deviation of mean sample-size-weighted correlations; SD_res_, residual standard deviation; SE $\bar{r}$, standard error of $\bar{r}$,; ρ, true-score correlation (corrected for unreliability in both measures for cases where artifact distributions are seen in Table 1); 90% CIρ, 90% confidence interval of ρ; 80% CVρ, 80% credibility values of ρ < 0.05. Because the studies that were determined as outliers in the correlation pools of demographic variables, it was deleted from the further analysis. Bold values indicate statistically significant meta correlation coefficients.

In the dispositional variables category, careerism [*r* = 0.29, ρ = 0.37, 90% CI (0.19, 0.55)], turnover intention [*r* = 0.34, ρ = 0.43, 90% CI (0.32, 0.55)], and moral disengagement [*r* = 0.33, ρ = 0.42, 90% CI (0.16, 0.67)] displayed notable and significant corrected true correlation with CCBs (90% CI did not contain zero, see Table 3).

In the skills category, political skill was found as unrelated to CCBs (90% include zero, see Table 3). On the other hand, in the job design category, job autonomy [*r* = −0.05, ρ = −0.06, 90% CI (−0.12, −0.01)] showed a small negative and significant correlation with CCBs.

Variables in the perceptions category displayed notable relations with CCBs. Specifically, felt obligation [*r* = 0.59, ρ = 0.75, 90% CI (0.74, 0.77)], work-family conflict [*r* = 0.57, ρ = 0.72, 90% CI (0.54, 0.90)], organizational-based self-esteem [*r* = 0.49, ρ = 0.62, 90% CI ( 0.31, 0.93)], abusive supervision [*r* = 0.32, ρ = 0.37, 90% CI (0.15, 0.60)], and feeling trusted [*r* = 0.38, ρ = 0.49, 90% CI (0.41, 0.58)] have a positive and significant true correlation with CCBs (90% CI did not include zero, see Table 3). Organizational identification [*r* = −0.24, ρ = −0.31, 90% CI (−0.56, −0.06)], leader-member exchange [*r* = −0.37, ρ = −0.48, 90% CI (−0.57, −0.38)], psychological safety [*r* = −0.28, ρ = −0.32, 90% CI (−0.40, −0.24)], distributive justice [*r* = −0.23, ρ = −0.30, CI (−0.58, −0.02)], and interactional justice [*r* = – 0.13, ρ = −0.15, CI (−0.20, −0.10)] displayed negative and notable relations with CCBs (90% CI did not include zero, see Table 3), while the climate for innovation and person-organization fit were not related to CCBs (90% CI include zero, see Table 4).

Organizational cynicism [*r* = 0.60, ρ = 0.77, 90% CI (0.65, 0.90)] and burnout [*r* = 0.55, ρ = 0.71, 90% CI (0.63, 0.79)] as employee attitudes were positively related to CCBs, while job satisfaction [*r* = −0.33, ρ = −0.43, 90% CI (−0.52, −0.33)], and organizational commitment [*r* = – 0.20, ρ = −0.26, 90% CI (−0.46, −0.06)] were negatively related to CCBs (90% CI did not include zero, see Table 3). On the other hand, the CCBs-trust in supervisor relationship was found as nonsignificant (90% CI including zero, see Table 4).

In the behaviors/outcomes sub-category, counterproductive workplace behaviors [*r* = 0.47, ρ = 0.60, 90% CI (0.33, 0.88)] displayed a positive and large relationship with CCBs. In contrast, facades of conformity [*r* = 0.37, ρ = 0.48, 90% CI (0.45, 0.51)] and social loafing [*r* = 0.24, ρ = 0.28, 90% CI (0.01, 0.55)] displayed a positive and moderate relationship with CCBs (90% CI did not include zero, see Table 3). On the other hand, organizational citizenship behaviors, job performance, and employee silence were not significantly related to CCBs (90% CI include zero, see Table 4).

Next, we examined the relationships between CCBs, and the variables categorized into feelings/emotions. Accordingly, work alienation [*r* = 0.50, ρ = 0.64, 90% CI (0.39, 0.90)], anger toward the organization [*r* = 0.41, ρ = 0.52, 90% CI (0.43, 0.61)], job stress [*r* = 0.32, ρ = 0.42, 90% CI (0.11, 0.72)], citizenship pressure (*r* = 0.36, ρ = 0.46, 90% CI (0.29, 0.64)] displayed positive and moderate relations with CCBs (90% CI did not include zero, see Table 3). On the other hand, negative affect and equity sensitivity were not related to CCBs (include zero, see Table 4). In sum, employees who are highly exposed to exhibit CCBs are more likely to feel negative feelings or emotions.

**Figure 7:**
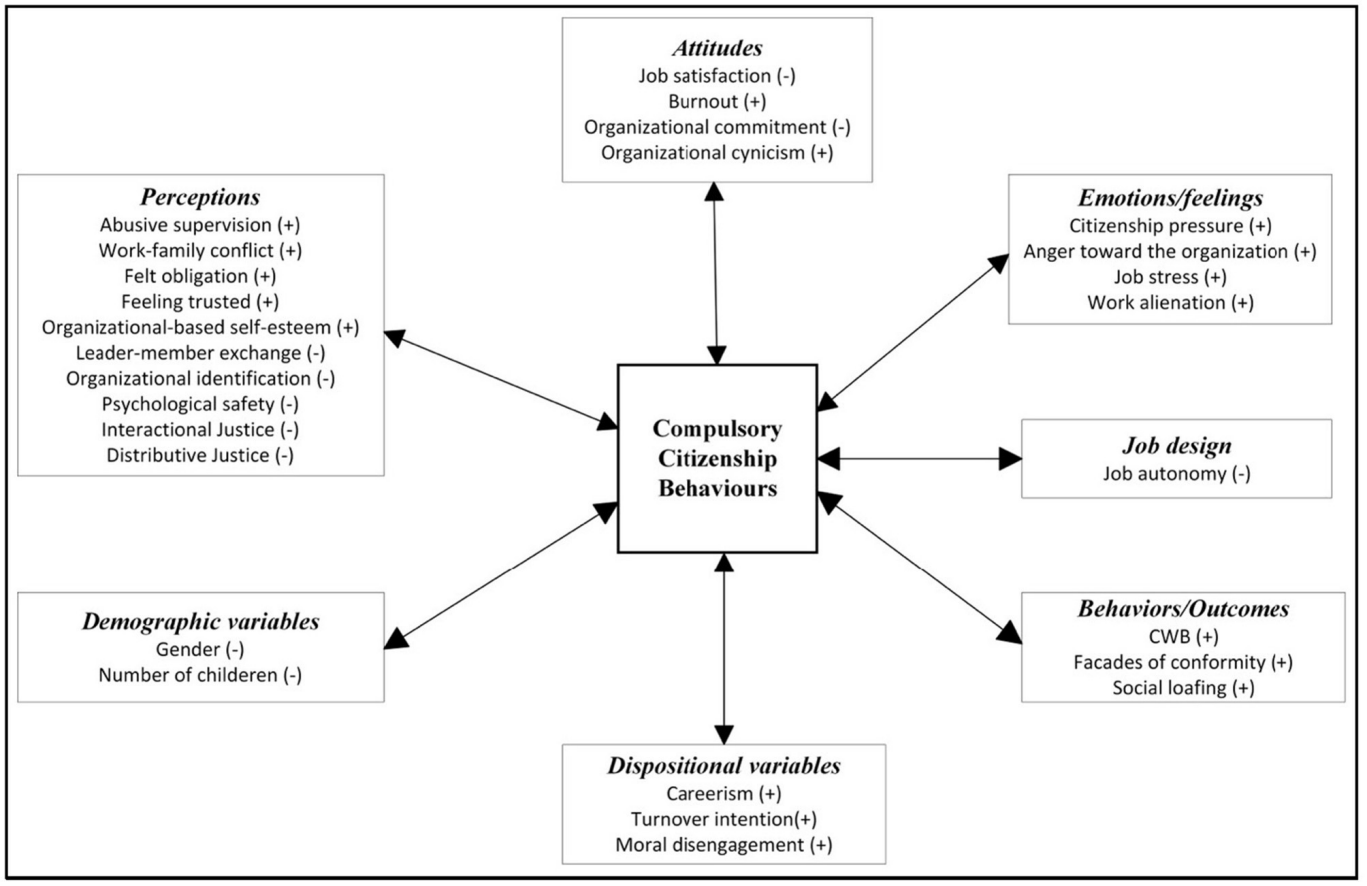
Cumulative results of significant correlates of CCBs.

In the cultural factors category, it was found that Chinese traditionality and S-s Guanxi were not related to CCBs (include zero, see Table 4). In sum, variables that are statistically significantly related to CCBs are presented with their direction in parentheses (see Figure 7).

## Discussion

This study is the first meta-analytic review of CCBs, synthesizing 180 correlations from 53 independent scientific publications in 2007–2022. When the countries of studies were evaluated, Turkey was in the first place, followed by China and Taiwan. Social exchange theory (SET), abusive supervision theory (AST), and conservation of resources theory (COR) were found as the most prefered theoretical lenses in the reviewed publications. This study offers several novel insights into the CCBs literature. Defining the overall effect sizes of the relations between CCBs and variables in the nomological network of CCBs is the methodologic contribution of this study. On the other hand, based on cumulative evidence, determining compulsory citizenship behaviors as undesirable and destructive behaviors for organizations is the theoretical contribution of this study.

### Demographic variables

Before discussing the results, Cohen's suggested effect sizes were taken as reference values to avoid a subjective assessment of effect sizes (i.e., 0.10 is small, 0.30 is moderate, and 0.50 is large) (Cohen, 2013).

Because there is some individual evidence about the relationship connection between CCBs and demographical characteristics (Zhao et al., 2014; Alkan, 2015; Liu et al., 2017; Liang, 2022; Liang et al., 2022), we also synthesized the correlations between CCBs and some demographical characteristic (e.g., age, gender, education, tenure, marital status, and work hours). Further, researchers need some evidence to include some factors that have the potential to affect their focus of interest as control variables in their research models (Luan et al., 2022). In this respect, a meta-analysis of the demographics plays an essential role in meta-analytic studies, and further empirical research needs this information.

Tatum (2022) pointed out that a US-based study conducted in 2017 indicated that female workers are more exposed to CCBs than men because of society's expectations (e.g., women are more prone to exhibit helping and communal behavior). Contrary to this evidence, our finding showed that men exhibit more CCBs than women, consistent with previous research (Zhao et al., 2014; Wang and Huang, 2019). This finding also overlaps with the findings of a meta-analytic study based on social role theory (Franke et al., 1997). Accordingly, the mentioned meta-analysis determined that women do not find various business practices ethical compared to men. Considering that CCBs are against employees' free will and positively associated with undesirable outcomes such as CWB (Su et al., 2021; Zuo, 2022), female employees may be less tolerant of exposure to this type of workplace behavior compared with male employees. Moreover, women and men have different motivations (Warr, 2008; Luan et al., 2022) because they have different social expectations. For example, working in a permanent paid job is more important for men than women (Warr, 1982, 2008). One of the reasons for this difference may be the competitiveness between the two. According to Cashdan (1998), in financial success, men are more competitive than women. Supporting this notion, most of the studies examined were conducted at the countries belonging to eastern culture (e.g., Turkey, China, Taiwan) where patriarchal culture is dominant (Ozaki and Otis, 2017). In these cultures, men may see themselves as if the hero of the family. In patriarchal culture, men may see themselves as the head of the family and take on the extra responsibility to deal with difficulties (e.g., CCBs). In patriarchal cultures, “men are more resilient than women, as breadwinners, at least men are considered more agile than women” (Eleanora and Supriyanto, 2020, p. 49). Thus, male employees can be exposed to CCBs more than female employees, and they can see the strength to cope with it.

The number of children was also found as the other related demographic variable with CCBs. The findings of previous studies are consistent with our cumulative results (Liu et al., 2017; Liang, 2022; Liang et al., 2022). The number of children is closely related to employees' family responsibilities. Bergeron (2007) emphasizes that employees with high flexibility in work-family balance, such as singles, are more likely to engage in discretionary behaviors. Supporting this notion, Bolino et al. (2010) hypothesized that family responsibilities (e.g., the number of children) have diminishing effects on the relationship between citizenship pressure and OCB. In other words, we infer these findings as managers or other powerful decision-makers in organizations are more likely to tolerate employees with more children in terms of CCB behaviors. Previous research also demonstrated that because single employees have fewer responsibilities, managers or supervisors tend to call these employees to make extra role demands (Flynn, 1996). In sum, it can be said that single employees have fewer family responsibilities and that managers primarily benefit from these employees for extra work demands. In this context, we suggest that future research on CCBs should include the number of children as a control variable in their models.

Our results indicated that gender and number of children display small but statistically significant relations with CCBs. Men and employees with fewer children display more CCBs than women and employees with more children. Based on our cumulative evidence, we suggest that future research include gender and the number of children as control variables in their research.

### Study variables

Because each included study has a different theoretical lens and because of the page and word limits of the journal, to understand the theoretical rationales of the relations among the variables, we recommend that readers, after presenting results with their citations below, check the relevant study's theoretical framework.

First, our results showed that turnover intention, moral disengagement, and careerism as dispositional variables are positively and moderately related to CCBs. These negative dispositions were explained by past research primarily considering the abusive supervision, social cognitive (SCT), and social exchange theories (SE) (Vigoda-Gadot, 2007; Yildiz and Ayaz Arda, 2018; He et al., 2019; Su et al., 2021). Although, most of these studies, which focus on the relationship between these dispositional variables and CCBs, see these variables as the triggers of harmful outputs (e.g., CCBs, Yildiz and Ayaz Arda, 2018; Dogan, 2019; CWB, Shu et al., 2018; turnover intention, Selcuk, 2017), some studies see CCBs as the resource consuming agent that awake employees to develop self-protection strategies (e.g., turnover intention, Yildiz and Elibol, 2021). However, the same study also emphasized that if there are insufficient employment conditions, the employee will not leave the job despite the intention to leave the job and may exhibit social loafing behaviors by withdrawing. Therefore, the dispositional variables associated with CCBs mainly result in negative results on employees. In addition, because these dispositional variables are associated with ethical cognition and ethic-related issues (Chiaburu et al., 2013; Demirtas and Akdogan, 2015; Ayaz Arda and Yildiz, 2019) and because SCT was used as the explanatory lens for the bases of these studies (Gizlier, 2018; Yildiz and Ayaz Arda, 2018), SCT should be considered as a critical theory in explaining the negative orientations/tendencies developed because of CCBs.

Second, we detected a small negative relationship between CCBs and job autonomy. Liu et al. (2017) explain this situation: employees with higher job autonomy have more flexibility in performing their jobs. Therefore, it can be said that these people take more responsibility than others. As these people are already accustomed to taking on more workloads, compared to employees with low job autonomy, they will be less bothered by their managers' extra work demands. They will not see these behaviors as CCBs. Although such a perception is because of autonomy, those with high autonomy should be given supportive feedback that such a compensatory mechanism is wrong and that they do not have such an obligation. The emergence of OCB based on the employees' wishes should be encouraged.

Although we determined a negative relationship between CCBs and political skills, this finding was not statistically significant, contrary to our expectations. Suppose political skills are necessary for influencing other people and being effective in the organization (Chen and Gao, 2020). In that case, employees are expected to act as if they are not disturbed by these behaviors by performing the extra tasks they are asked to do politically. In other words, it can be said that employees with these abilities may appear as if they are doing the CCBs imposed on them wholeheartedly, or they deliberately sacrifice themselves to do extra tasks today so as not to lose potential gains in the future (Shu et al., 2018). Supporting this notion, Bromley (1993) emphasized that employees with high political skills also have a higher level of self-efficacy. The low number of synthesized studies (e.g., for job autonomy) may be another convincing explanation for this situation. Hunter and Schmidt (2004) explain this situation: When number of studies too small, the confidence interval widens when the error variance increases, so the confidence interval is more likely to contain zero. Therefore, further individual studies are recommended for future researchers to provide more robust evidence to test whether this relationship between political skill and CCBs is significant. In addition, because individual studies examine the role of political skill as a kind of impression management tactic (e.g., Shu et al., 2018; Chen and Gao, 2020), further research should consider this factor as a control variable with the potential to influence their results.

Third, in the perceptions category, except for the climate for innovation and person-organization fit, all synthesized perceptions were significantly associated with CCBs. Felt obligation, work-family conflict, and organizational-based self-esteem are largely and positively related to CCBs while feeling trusted and abusive supervision are moderately and positively related to CCBs. On the other hand, leader-member exchange (LMX), psychological safety, organizational identification, and distributive justice are negatively and moderately related to CCBs. In contrast, interactional justice displays a small and significant negative relationship with CCBs.

According to social exchange theory (SET) (Blau, 1964), employees and organizations interact to maximize their rewards and minimize costs (Homans, 1958; Salam et al., 1998; Shiau and Luo, 2012). When above mentioned perceptional variables are evaluated, it can be said that most of them are the antecedents and consequences of CCBs based on the reciprocity principle stated in the SET. The findings in organizational behavior research show that interpersonal social interactions affect individuals' perceptions and long-term contracts. Therefore, interdependence will continue until a consistent and satisfactory exchange between these changes occurs (Homans, 1958). In this context, it can be said that studies examining the relationships between CCBs, and perceptions are grouped into three sub-categories, namely “from CCBs to negative perceptions,” “from negative perceptions to CCBs,” and “from positive perceptions to CCBs.” Studies in the first group (from CCBs to negative perceptions) refer to employees who are uncomfortable with the extra-role behaviors imposed on them and develop negative perceptions toward the organization as a response to these behaviors (e.g., work-family conflict, interactional and distributive justice, organizational identification). The second group (from negative perceptions to CCBs) refers to employees who exhibit CCBs due to various negative perceptions (e.g., abusive supervision). The third group (from positive perceptions to CCBs) expresses that employees feel obliged to do non-duty tasks because of normative pressure (e.g., feeling trusted, felt obligation, organizational-based self-esteem, organizational identification, leader-member exchange). Given these explanations, it can be asserted that researchers intending to investigate CCBs ought to take into account these three perspectives while developing their theoretical models founded on SET. Notably, the third group is susceptible to being concealed, thus hindering a clear determination of whether OCB is voluntary or coerced.

On the other hand, a plausible explanation for the non-significant relations between CCBs and climate for innovation and CCBs and person-organization fit may be attributed to the insufficient number of synthesized studies (Hunter and Schmidt, 2004). We encourage further research to retest our results with more individual studies. After enough studies accumulated regarding the mentioned relations, an updated meta-analysis could provide more accurate evidence for our findings.

Fourth, in the attitudes category, except for trust in the supervisor, all employee attitudes significantly correlated with CCBs. Specifically, organizational cynicism and burnout are largely and positively related to CCBs. It can be said that CCBs are strongly associated with these attitudinal variables. On the other hand, while job satisfaction is moderately and negatively associated with CCBs, organizational commitment shows a small relation to CCBs. Because all these employee attitudes are primarily identified based on SET (e.g., Peng and Zhao, 2012; Tabuk, 2016; Bozdogan, 2022) and COR (e.g., He et al., 2018; Telli Danismaz, 2021), we concluded that SET and COR are useful theoretical lenses that should be considered when explaining attitudinal consequences of CCBs. From the SET perspective, employees may develop low levels of commitment and job satisfaction and high levels of burnout and cynicism in response to CCB exposure.

On the other hand, the COR prospective studies focused on the resource consumption effect of CCBs on employee attitudes. Such studies consider the COR lens to explain the passive reactions of employees to protect themselves from CCB's harmful effects (He et al., 2018; Telli Danismaz, 2021; Yildiz and Elibol, 2021). In addition, as mentioned earlier, the non-significant relationship between CCBs and trust in supervisor may stem from insufficient studies.

Fifth, in the behaviors category, all other variables except for the counterproductive workplace behaviors (CWB), social loafing, and facades of conformity were insignificantly related to CCBs. In sum, these findings signify that forcing employees to engage in CCBs leads to social loafing behaviors and CWB that can cause significant productivity losses rather than benefit the organization. The positive relationship among CCBs, CWB, and social loafing behaviors was considered a noteworthy finding that CCB is a variable with devastating effects on organizations. There has not been a common theoretical perspective explaining the relationship between CCBs and CWB because of the scarcity of studies (e.g., affective events theory, Zuo, 2022; SET, Su et al., 2021; equity theory, Guarino, 2016). However, the common point of these theoretical lenses is that employees exhibit CWB and social loafing behaviors in response to dissatisfaction and negative emotions (e.g., anger toward the organization, Che, 2015; Yildiz et al., 2022a). The reason for these reactions is the employees' disappointments arising from fairness perception and social exchange interactions within the organization. As a result, future research should focus on theories explaining the emotions that play a crucial role in CCBs causing CWB and social loafing behaviors.

On the other hand, based on affective events theory, studies also showed that facades of conformity as a kind of self-misrepresentation strategy emerge when employees want to ensure their comfort within the organization after being exposed to exhibit CCBs (Liang, 2022; Liang et al., 2022). Employees may not seem bothered by the extra duties imposed by the organization or the manager. However, facades of conformity are associated with many negative variables (e.g., turnover intention, Hewlin, 2009; lower levels of job satisfaction, Chou et al., 2020, lower levels of work engagement, Hewlin et al., 2017). Furthermore, it is quite difficult to measure the negative consequences of CCBs behaviors in those who display facades of conformity. Future researchers should include misrepresentation strategies such as facades of conformity into their models as control variables to access more accurate information in research on CCBs.

As earlier mentioned, relations of employee silence, social loafing, OCB, and job performance with CCBs were found as nonsignificant. The number of studies in the meta-analysis is small for employee silence. Therefore, to reach more accurate knowledge on this relationship accumulation of individual studies is required. For social loafing behaviors, despite individual studies found a positive relationship between CCBs and social loafing behaviors (Gizlier, 2018; Yakin and Sokmen, 2018; Yildiz and Elibol, 2021), the overall effect size of this relationship was not found as significant. One plausible explanation for this result might be that employees reported less social loafing behaviors because of facades of conformity, social desirability bias (Yildiz and Elibol, 2021), and managerial pressure. Still, they tend to display more social loafing behaviors. For job performance, a possible explanation is that employees forced to display CCBs tend to underperform or withdraw contribution to the organization's well-being (Ahmadian et al., 2017; Topcu et al., 2017; Hung et al., 2022). However, they may still report as if they are performing more. The non-significant OCB-CCBs relationship is another remarkable finding of this study. This finding suggests that CCBs are a distinct construct that is not related to OCB. Therefore, it can be posited that CCBs should not be considered as the negative form of OCB. Consequently, utilizing this finding as a benchmark for future research or conducting further investigations will bolster the understanding of whether CCBs are indeed a unique construct. Additionally, this approach will unveil other antecedents and outcomes that have not yet been discovered, highlighting the different perspectives of this distinct structure.To access more accurate information about the discussed relations above, future researchers should include other variables that could affect the examined variables in their models as control or moderator variables. Next, new meta-analyses should re-synthesize the studies that will accumulate in this direction.

Sixth, in the feelings/emotions category, while work alienation and anger toward the organizations are largely and positively related to CCBs, citizenship pressure and job stress display a moderate and positive relationship with CCBs. On the other hand, the relations of negative affect and equity sensitivity with CCBs were found as non-significant. Citizenship pressure based on the social exchange theory was used as a predictor of CCBs (Vigoda-Gadot, 2006). The relations of work alienation, job stress, and anger toward organizations with CCBs based on SET were explained as employees' reactions to CCBs (Tabuk, 2016; Kerse et al., 2019; Surucu, 2019). Considering the strong relationship of CCBs with anger and alienation and relationships with many undesirable employee behaviors (e.g., CWB, Zuo, 2022; turnover intention and burnout, Ozturk Ciftci, 2021; lower levels of job satisfaction and job involvement, Chiaburu et al., 2014), future researchers should determine what kind of consequences the emotional variables originating from CCBs cause. Based on these explanations and most of the studies' usage, SET should be considered as a useful theoretical lens for explaining the emotional correlates of CCBs. As previously mentioned, the non-significant relations between negative affect and equity sensitivity may be attributed to a small number of studies included in this study. Because few studies in meta-analysis cause a wide range of confidence intervals, zero is likely to be in the confidence interval. Future research should more sensitively consider this issue and evaluate the correlations more accurately.

Finally, in the culture category, the relations of Chinese traditionality (*k* = 2) and S-s Guanxi (*k* = 2) with CCBs were not statistically significant. To reach more robust knowledge, more empirical research should be conducted to correct sampling errors effectively and, in turn, to reach more accurate results.

## Conclusion

Scholars have made a significant effort to develop the CCBs literature in the past two decades. This first meta-analytic review outlined the findings of the quantitative studies conducted on CCBs. Our results show that CCBs are a common phenomenon, especially in eastern cultures (e.g., Turkey, China, and Taiwan). Further, male workers and employees with fewer children display more CCBs. Our results also indicated that turnover intention, moral disengagement, careerism, felt obligation, work-family conflict, organizational-based self-esteem, abusive supervision, feeling trusted, organizational cynicism, burnout, counterproductive workplace behaviors, facades of conformity, work alienation, anger toward the organization, citizenship pressure, and job stress are positively related to CCBs. We also found that job autonomy, leader-member exchange, psychological safety, organizational identification, job satisfaction, and organizational commitment are negatively associated with CCBs. We believe that our findings provide solid cumulative evidence for the nomological network of CCBs. We expect that this meta-analysis will contribute to the development of CCBs literature.

### Limitations and future research

Despite its robust analytical nature, this meta-analysis is not without its limitations. Firstly, because we synthesized correlation coefficients of the included publications to dermine overal effectsizes and their directions, causal effects among the variables were not investigated. In addition, there is a need to increase the number of available quantitative studies to test causal effects and arrive at robust findings (e.g., *k* ≥ 5, *k* ≥ 10) (Borenstein et al., 2009; Higgins and Green, 2011). Therefore, this study does not have findings to express causal effects such as antecedents or consequences of the CCBs. Further research should contribute to the current research pool by testing studied relationships in different settings or research designs. Secondly, because one inclusion criterion is about the publications' language, English and Turkish, we recommend further research to conduct another version of this study, including the studies published in other languages. Third, because there are limited studies in the literature, some of the investigated relations were found non-significant. It is necessary to accumulate enough studies to reach a more precise judgment non-significant relations mentioned above.

As emphasized earlier, positive variables that related to CCBs (e.g., felt obligation, feeling trusted, organizational-based self-esteem) is a new research avenue in CCBs literature. For instance, a recent study showed that CCBs result from feeling trusted (Wang and Huang, 2019). Although feeling trusted facilitates positive outcomes such as performance and OCBs, the trust-based relations between supervisors and subordinates cause employees to feel “responsible and ownership” (Wang and Huang, 2019). Based on the principle of reciprocity in social exchange theory, feeling trusted by a supervisor causes more workload and avoidance of reputation loss (Baer et al., 2015). The positive relationships between the CCBs and above mentioned variables can be explained by various theoretical perspectives. In the context of Self-Determination Theory (Deci and Ryan, 2000; Ryan and Deci, 2000), it can be argued that trusting employees, having high organizational-based self-esteem, and having high levels of self-efficacy satisfy employees' competence, autonomy, and relatedness needs, leading them to perceive tasks outside of their job descriptions as their own tasks and thus becoming more competent employees. On the other hand, according to Broaden and Build Theory (Fredrickson, 2001), positive emotions enable employees to broaden their cognitive, behavioral, social, ad psychological resources, thereby engaging in tasks that are not explicitly included in their job responsibilities. As a result of positive emotional experiences and positive affect, employees may develop a sense of engagement with their organization based on positive emotions such as gratitude (Lyubomirsky et al., 2005). This dedication, in turn, may motivate employees to perform non-work-related tasks beyond their job requirements. From the lens of the Conservation of Resources Theory (Hobfoll, 1989), people try to retain and protect resources such as an object, personal characteristics, conditions, and energy, which people value (Halbesleben et al., 2009). People tend to avoid factors they think will put these resources at risk. Therefore, the loss or reduction of these resources constitutes the primary concern. However, positive perceptions, attitudes, and behaviors, on the contrary, may cause the existing positive resources to increase even more. Therefore, employees can allocate the surplus of these positive resources to doing work that is not their duty. Further, in addition to the above mentioned theoretical perspectives, the Self-Efficacy Theory (Bandura, 1997) may be another theoretical lens to explain the positive link between CCBs and positive variables. According to this theory, employees who possess a high level of self-efficacy are more likely to develop effective coping strategies in response to difficulties. Self-efficacy explains employees' ability to perform tasks beyond their normal duties and sustain them over time. As a result, employees with high self-efficacy are anticipated to exhibit increased levels of extra-role behaviors or performance (Stajkovic and Luthans, 1998; Salanova et al., 2011).

In light of the above mentioned explanataions, both positive antecedents of CCBs can be identified, and the general acceptance of the concept arising from managerial or social pressure may need to be updated. For example, because the consciousness trait is related to many positive variables, such as organizational-based self-esteem (Hahn and Mathews, 2022), and negative consequences, such as presenteeism (Yildiz et al., 2017), this trait may also be one of the potential predictors of CCBs. Similarly, as Tatum (2022) points out, while work engagement is positively associated with productivity and performance, it can also lead to CCBs. Individual efforts to explore these new research areas and accumulate findings in this direction will provide a comprehensive understanding and management of the CCBs.

On the other hand, when the reviewed studies are evaluated, although studies show that abusive supervision and authoritarian leadership are positively associated with CCBs (Shu et al., 2018; Dogan, 2019; Wang and Huang, 2019), studies showing which leadership styles are negatively correlated with CCBs are insufficient. Although Yildiz and Yildiz (2016) proposed that ethical leadership, servant leadership, and LMX may diminish CCBs, and we shared He et al.'s (2019, p. 271) prediction that “ethical leadership, benevolent leadership, and servant leadership will lead to fewer CCBs”, we could not conduct a meta-analysis on these types of leadership because there was not enough empirical publication in the current literature. We suggest for future researchers to investigate the effects of the leadership styles mentioned on CCBs. Finally, the publications reviewed in this study were primarily conducted in countries where eastern culture is dominant. More empirical studies in western culture are needed to test the generalizability of the results.

## Data availability statement

The original contributions presented in the study are included in the article/Supplementary material, further inquiries can be directed to the corresponding author.

## Author contributions

BY, EE, and ZK contributed to the conception and design of the study. BY, ZK, EE, and TY organized the database. BY performed the statistical analysis and wrote the first draft of the manuscript. BY, ZK, HY, and MO wrote sections of the manuscript. All authors contributed to the manuscript revision, read, and approved the submitted version.
