# Supplementary material for: A systematic review and meta-analytic synthesis of the relationship between compulsory citizenship behaviors and its theoretical correlates
Source: Front Psychol. 2023 May 4;14:1120209. doi: 10.3389/fpsyg.2023.1120209 (PMC10192750; doi:10.3389/fpsyg.2023.1120209)
Supplement: Supplementary file 1 [file Table_1.docx]

Supplementary Data

## 1 - Included Studies Summary

| **Author/Year** | **Country** | **Sample Size** | **Sector** | **Type** | **Study Design** | **Outliers Management** | **Scale** | **Reliability (α)** |
| --- | --- | --- | --- | --- | --- | --- | --- | --- |
| Ahmadian et al. (2017) | Turkey | 635 | Accommodation | Published | Cross-sectional | No | Vigoda-Gadot, 2007 | 0.940 |
| Alkan (2015) | Turkey | 170 | Finance | Unpublished | Cross-sectional | No | Organ and Konovsky, 1989, 1996; Bolino et.al, 2010 Vigoda-Gadot, 2007 | 0.943 |
| Bashir et al. (2019) | Pakistan | 368 | University | Published | Cross-sectional | No | Vigoda-Gadot, 2007 | 0.880 |
| Can & Begenirbas (2020) | Turkey | 175 | Academicians & Teachers | Published | Cross-sectional | Yes | Vigoda-Gadot, 2007 | 0.892 |
| Che (2015) | USA | 71 | Registered nurses | Unpublished | Cross-sectional | No | Vigoda-Gadot, 2007 | 0.700 |
| Chen & Gao (2020) | China | 298 | Employees | Published | Cross-sectional | No | Vigoda-Gadot, 2007 | 0.652 |
| Chen et al. (2021) | China | 505 | High-tech  enterprises | Published | Cross-sectional | No | Vigoda-Gadot, 2007 | 0.800 |
| Coban (2021) | Turkey | 305 | Employees | Published | Cross-sectional | No | Vigoda-Gadot, 2007 | 0.918 |
| Dogan (2019) | Turkey | 278 | Employees | Unpublished | Cross-sectional | No | Vigoda-Gadot, 2007 | 0.901 |
| Gizlier (2018) | Turkey | 334 | Service sector | Unpublished | Longitudinal | Yes | Vigoda-Gadot, 2007 | 0.910 |
| Guarino (2016) | USA | 147 | NA | Unpublished | Longitudinal | Yes | Vigoda-Gadot, 2007 | 0.960 |
| Gursoy & Koksal (2018) | Turkey | 192 | Employees | Published | Cross-sectional | No | Vigoda-Gadot, 2007 | 0.741 |
| He et al. (2018) | China | 242 | Manufacturing | Published | Longitudinal | No | Vigoda-Gadot, 2007 | 0.800 |
| He et al. (2020) | China | 251 | Manufacturing | Published | Longitudinal | No | Vigoda-Gadot, 2007 | 0.790 |
| He et al. (2019) | China | 293 | Manufacturing | Published | Longitudinal | No | Vigoda-Gadot, 2007 | 0.770 |
| Jin & Hahm (2019) | China | 175 | IT workers | Published | Cross-sectional | No | Vigoda-Gadot, 2007 | 0.940 |
| Kerse et al. (2019) | Turkey | 104 | Employees | Published | Cross-sectional | No | Vigoda-Gadot, 2007 | 0.964 |
| Kocak (2018) | Turkey | 297 | Manufacturing Employees | Published | Cross-sectional | No | Vigoda-Gadot, 2007 | 0.930 |
| Koksal (2020) | Turkey | 227 | Hotel employees | Published | Cross-sectional | No | Vigoda-Gadot, 2007 | 0.947 |
| Liang (2022) | Taiwan | 356 | Retail and distribution corporation | Published | Longitudinial | No | Vigoda-Gadot, 2007 | 0.830 |
| Liang et al. (2022) | Taiwan | 655 | Banking | Published | Longitudinal | No | Vigoda-Gadot, 2007 | 0.830 |
| Peng & Zhao (2011) | China | 450 | Supervisor - subordinates | Published | Cross-sectional | No | Vigoda-Gadot, 2007 | 0.835 |
| Pradhan & Gupta (2021) | India | 188 | NA | Published | Cross-sectional | No | Vigoda-Gadot, 2007 | 0.903 |
| Selcuk (2017) | Turkey | 191 | Nurses | Unpublished | Cross-sectional | No | Vigoda-Gadot, 2007 | 0.805 |
| Shu et al. (2018) | Taiwan | 237 | Manufacturing, service  and finance | Published | Longitudinal | No | Vigoda-Gadot, 2007 | 0.890 |
| Su et al. (2021) | China | 525 | Manufacturing | Published | Cross-sectional | No | Vigoda-Gadot, 2007 | 0.856 |
| Surucu (2019) | KKTC | 302 | Hotel employees | Unpublished | Cross-sectional | No | Vigoda-Gadot, 2007 | 0.880 |
| **Author/Year** | **Country** | **Sample Size** | **Sector** | **Type** | **Study Design** | **Outliers Management** | **Scale** | **Reliability (α)** |
| Tabuk (2016) | Turkey | 301 | Academicians | Unpublished | Cross-sectional | No | Vigoda-Gadot, 2007 | 0.870 |
| Telli (2021) | Turkey | 374 | White-collar workers | Unpublished | Cross-sectional | No | Vigoda-Gadot, 2007 | 0.830 |
| Topcu et al. (2017) | Turkey | 138 | Employees | Published | Cross-sectional | Yes | Vigoda-Gadot, 2007 | 0.834 |
| Tuzgel (2021) | Turkey | 385 | Employees | Unpublished | Cross-sectional | No | Vigoda-Gadot, 2007 | 0.895 |
| Unaldi Baydin et al. (2020) | Turkey | 569 | Hospital | Published | Longitudinal | No | Vigoda-Gadot, 2007 | 0.860 |
| Vigoda-Gadot (2007) | Israel | 206 | Education | Published | Cross-sectional | No | Vigoda-Gadot, 2007 | 0.840 |
| Wang & Huang (2019) | China | 509 | Healthcare-nurses | Published | Cross-sectional | No | Vigoda-Gadot, 2007 | 0.958 |
| Wang & Huang (2019) | China | 349 | Hotel employees | Published | Longitudinal | No | Vigoda-Gadot, 2007 | 0.834 |
| Watters (2012) | UK | 119 | Banking | Unpublished | Cross-sectional | No | Vigoda-Gadot, 2007 | 0.850 |
| Wu et al. (2018) | China | 324 | NA | Published | Longitudinal | No | Vigoda-Gadot, 2007 | 0.880 |
| Yakin & Sokmen (2018) | Turkey | 160 | Employees | Published | Cross-sectional | No | Vigoda-Gadot, 2007 | 0.910 |
| Yildiz & Ayaz Arda (2018) | Turkey | 330 | Bankers | Published | Cross-sectional | No | Vigoda-Gadot, 2007 | 0.760 |
| Yildiz & Elibol (2021) | Turkey | 264 | Nurses | Published | Cross-sectional | Yes | Vigoda-Gadot, 2007 | 0.880 |
| Liu et al. (2017) | China | 312 | NA | Published | Longitudinal | No | Vigoda-Gadot, 2007 | 0.870 |
| Zhao et al. (2013) | China | 434 | Service | Published | Cross-sectional | No | Vigoda-Gadot, 2007 | 0.940 |
| Zhao et al. (2014) | China | 388 | NA | Published | Longitudinal | No | Vigoda-Gadot, 2007 | 0.870 |
| Bozdogan (2022) | Turkey | 432 | Textile | Published | Cross-sectional | No | Vigoda-Gadot, 2007 | 0.930 |
| Celik & Ongel (2022) | Turkey | 398 | University | Published | Cross-sectional | Yes | Vigoda-Gadot, 2007 | 0.890 |
| Coban (2022) | Turkey | 361 | Retail and distribution corporation | Published | Cross-sectional | Yes | Vigoda-Gadot, 2007 | 0.917 |
| Eivazzadeh & Nadiri (2022) | Iran | 925 | University | Published | Cross-sectional | No | Vigoda-Gadot, 2007 | 0.842 |
| He et al. (2022) | China | 227 | NA | Published | Longitudinal | Yes | Vigoda-Gadot, 2007 | 0.874 |
| Hung et al. (2022) | Taiwan | 393 | Hotel employees | Published | Cross-sectional | Yes | Vigoda-Gadot, 2007 | NA |
| Lin & Chi (2022) | Taiwan | 276 | NA | Published | Longitudinal | Yes | Vigoda-Gadot, 2007 | 0.870 |
| Sajuyigbe et al. (2022) | Nigeria | 420 | University | Published | Cross-sectional | No | Vigoda-Gadot, 2007 | 0.812 |
| Zuo (2022) | China | 312 | NA | Unpublished | Cross-sectional | Yes | Vigoda-Gadot, 2007 | 0.903 |
| Liu et al. (2019) | China | 298 | NA | Published | Cross-sectional | No | Vigoda-Gadot, 2007 | 0.740 |
| Yildiz et al. (2022) | Turkey | 294 | Healthcare-nurses | Published | Cross-sectional | Yes | Vigoda-Gadot, 2007 | 0.910 |

## 2 - Included Studies by Variables

| ***Variable*** | ***k*** | ***Studies*** |
| --- | --- | --- |
| *Dispositional variables* |  |  |
| Turnover Intention | 12 | Ahmadian et al., 2017; Can & Begenirbas, 2020; Celik & Ongel, 2022; Eivazzadeh & Nadiri, 2022; Gizlier, 2018; Jin & Hahm, 2019; Sajuyigbe et al., 2022; Selcuk, 2017; Tabuk, 2016; Vigoda-Gadot, 2007; Yildiz & Ayaz, 2018; Yildiz & Elibol, 2021 |
| Moral Disengagement | 3 | He et al., 2019; Su et al., 2021; Yildiz et al., 2021 |
| Careerism | 3 | Doğan, 2019; Gizlier, 2018; Yıldız & Ayaz Arda, 2018 |
| *Ability/Skill* |  |  |
| *Political Skill* | 2 | Chen & Gao, 2020; Shu et al., 2018 |
| *Job design* |  |  |
| Job Autonomy | 2 | Liu et al., 2017; Vigoda-Gadot, 2007 |
| *Perceptions* |  |  |
| Felt Obligation | 2 | Wang & Huang, 2019; Wang & Huang, 2019 |
| Work-Family Conflict | 4 | Chen et al., 2021; Koksal, 2020; Liu et al., 2017; Pradhan & Gupta, 2021 |
| Org. Based Self Esteem | 2 | Wang & Huang, 2019; Wang & Huang, 2019 |
| Abusive Supervision | 4 | Dogan, 2019; Pradhan & Gupta, 2021; Wang & Huang, 2019; Zhao et al., 2013 |
| Feeling Trusted | 2 | Wang & Huang, 2019; Wang & Huang, 2019 |
| Person-Organization Fit | 2 | Kocak, 2018; Tabuk, 2016 |
| Leader-Member Exchange | 6 | Celik & Ongel, 2022; Gursoy & Koksal, 2018; Kocak, 2018; Lin & Chi, 2022; Surucu, 2019; Tuzgel, 2021 |
| Psychological Safety | 3 | Alkan, 2015; Eivazzadeh & Nadiri, 2022; Zhao et al., 2013 |
| Organizational Identification | 3 | He et al., 2018; Tuzgel, 2021; Zhao et al., 2014 |
| Climate for Innovation | 3 | Ahmadian et al., 2017; Surucu, 2019; Vigoda-Gadot, 2007 |
| Distributive Justice | 2 | Eivazzadeh & Nadiri, 2022; Guarino, 2016 |
| Interactional Justice | 2 | Eivazzadeh & Nadiri, 2022; Zhao et al., 2014 |
| *Attitudes* |  |  |
| Organizational Cynicism | 4 | Coban, 2021; Coban, 2022; Topcu et al., 2017; Yakin & Sokmen, 2018 |
| Burnout | 10 | Ahmadian et al., 2017; Bozdogan, 2022; Che, 2015; He et al., 2018; Jin & Hahm, 2019; Liang et al., 2022; Pradhan & Gupta, 2021; Su et al., 2021; Tabuk, 2016; Vigoda-Gadot, 2007 |
| Trust in Supervisor | 2 | Wang & Huang, 2019; Wang & Huang, 2019 |
| Job Satisfaction | 6 | Che, 2015; Kerse et al., 2019; Topcu et al., 2017; Vigoda-Gadot, 2007; Watters, 2012; Zuo, 2022 |
| Organizational Commitment | 3 | Che, 2015; Peng & Zhao, 2011; Telli, 2021 |
| *Behaviors/Outcomes* |  |  |
| Employee Silence | 2 | He et al., 2018; Su et al., 2021 |
| CWB | 3 | Guarino, 2016; Su et al., 2021; Zuo, 2022 |
| Facades of Conformity | 2 | Liang, 2022; Liang et al., 2022 |
| Social Loafing | 3 | Gizlier, 2018; Yakin & Sokmen, 2018; Yildiz & Elibol, 2021 |
| OCB | 5 | Lin & Chi, 2022; Vigoda-Gadot, 2007; Wang & Huang, 2019; Wang & Huang, 2019; Zhao et al., 2014 |
| Job Performance | 4 | Ahmadian et al., 2017; Hung et al., 2022; Topcu et al., 2017; Zuo, 2022 |
| *Feelings/ Emotions* |  |  |
| Work Alienation | 2 | Tabuk, 2016; Tuzgel, 2021 |
| Anger toward organization | 2 | Che, 2015; Yildiz et al., 2022 |
| Citizenship Pressure | 2 | Liang, 2022; Liu et al., 2017 |
| Job Stress | 8 | Ahmadian et al., 2017; Chen et al., 2021; Guarino, 2016; Kerse et al., 2019; Surucu, 2019; Unaldi Baydin et al., 2020; Vigoda-Gadot, 2007; Watters, 2012 |
| Negative Affect | 3 | Bashir et al., 2019; He et al., 2018; He et al., 2020 |
| Equity Sensitivity | 3 | Guarino, 2016; Lin & Chi, 2022; Shu et al., 2018 |
| *Chinese Culture* |  |  |
| Chinese Traditionality | 2 | Peng & Zhao, 2011; Zhao et al., 2013 |
| S-s Guanxi | 2 | He et al., 2019; Wu et al., 2018 |
